# Integrative analysis of transcriptome and target metabolites uncovering flavonoid biosynthesis regulation of changing petal colors in *Nymphaea* ‘Feitian 2’

**DOI:** 10.1186/s12870-024-05078-5

**Published:** 2024-05-07

**Authors:** Xian Zhou, Xiaohan Wang, Haohui Wei, Huijin Zhang, Qian Wu, Liangsheng Wang

**Affiliations:** 1 State Key Laboratory of Plant Diversity and Specialty Crops, Institute of Botany, Chinese Academy of Sciences, Beijing, 100093 China; 2 China National Botanical Garden, Beijing, 100093 China; 3 University of Chinese Academy of Sciences, Beijing, 100049 China; 4 Hunan Agricultural University, Changsha, 410128 China

**Keywords:** *Nymphaea* ‘Feitian 2’, Transcriptome, Flavonoid, Color change

## Abstract

**Background:**

*Nymphaea* (waterlily) is known for its rich colors and role as an important aquatic ornamental plant globally. *Nymphaea atrans* and some hybrids, including *N*. ‘Feitian 2,’ are more appealing due to the gradual color change of their petals at different flower developmental stages. The petals of *N.* ‘Feitian 2’ gradually change color from light blue-purple to deep rose-red throughout flowering. The mechanism of the phenomenon remains unclear.

**Results:**

In this work, flavonoids in the petals of *N.* ‘Feitian 2’ at six flowering stages were examined to identify the influence of flavonoid components on flower color changes. Additionally, six cDNA libraries of *N*. ‘Feitian 2’ over two blooming stages were developed, and the transcriptome was sequenced to identify the molecular mechanism governing petal color changes. As a result, 18 flavonoid metabolites were identified, including five anthocyanins and 13 flavonols. Anthocyanin accumulation during flower development is the primary driver of petal color change. A total of 12 differentially expressed genes (DEGs) in the flavonoid biosynthesis pathway were uncovered, and these DEGs were significantly positively correlated with anthocyanin accumulation. Six structural genes were ultimately focused on, as their expression levels varied significantly across different flowering stages. Moreover, 104 differentially expressed transcription factors (TFs) were uncovered, and three *MYBs* associated with flavonoid biosynthesis were screened. The RT-qPCR results were generally aligned with high-throughput sequencing results.

**Conclusions:**

This research offers a foundation to clarify the mechanisms underlying changes in the petal color of waterlilies.

**Supplementary Information:**

The online version contains supplementary material available at 10.1186/s12870-024-05078-5.

## Background

Flower color is a remarkable and diverse characteristic throughout the plant kingdom. It not only plays a vital role in the success of plant survival or reproduction but also has significant economic benefits for the flower industry [1, 2]. Some factors influencing the development of flower color encompass the classes and levels of pigments in the petals, co-pigmentation effects, cell vacuole pH, petal epidermal cell structure, and complexes with metal ions [3]. Among these, the type and contents of pigments in the petals exert the greatest influence [4]. Anthocyanins, a class of flavonoids, influence 80% of angiosperms flower colors globally, including yellow, pink, red, purple, and blue [5–7].

In addition to single-color flowers, some plants possess complex flower color patterns. Some flowers can gradually change color over the course of the flowering period, including *Lonicera japonica* (white to gold), *Xanthoceras sorbifolium* (yellow or orange to red), *Lantana camara* (yellow to orange to scarlet to magenta), *Brunfelsia acuminata* (deep purple to light purple to white), *Hibiscus mutabilis* (white to pink to red), *Combretum indicum* (white to red), and *Victoria* (white to pink or ruby red). The color change mechanism varies between different flowers. The color change of *L. japonica* flowers was caused by β-carotene concentrations increasing dramatically in the white to golden flower stages, accompanied by a drop in lutein concentrations [8]. In *B. acuminate*, the anthocyanin content decreased, and the petal color changed [9]. The alteration in petal coloration has been attributed to an elevated concentration of cyanidin derivatives or delphinidin derivatives in the vacuoles of petals [10–14]. The molecular mechanisms underlying color changes are receiving increasing attention. In *Paeonia* ‘Coral Sunset’, the change in flower color from coral to pink to pale yellow is due to a significant decrease in anthocyanin content. Then, eight structural genes related to anthocyanin synthesis were highly expressed during the S1 period and lowly expressed during other stages, causing petal color changed [15]. In *Nelumbo* ‘Qiusanse’, petals color faded during flowering. Researchers have found that the involvement of anthocyanin biosynthesis repressors and degrading genes as well as pH regulators in controlling color fading [16].

Waterlilies, a general term for plants in the *Nymphaea* genus, are perennial aquatic plants with high ornamental, cultural, and economic value in the family Nymphaeaceae [17–19]. They are also the national flower of Egypt, Thailand, and other countries. Waterlilies represent an early diverging clade of flowering plants with unique roles in angiosperm phylogeny [20–22]. They are the most diverse and widespread genus of the family Nymphaeaceae, including five subgenera, including *Lotos*, *Hydrocallis*, *Anecphya*, *Brachyceras*, and *Nymphaea* [23, 24]. Waterlilies are globally distributed, with over 50 species, including abundant germplasm resources, with over 1000 cultivars globally.

Waterlilies display various flower colors, including white, yellow, red, purple, and blue, among others. Flavonoids are the primary pigments responsible for floral color in waterlilies. To date, 117 flavonoids have been identified in waterlilies, including 20 anthocyanins [25]. In examining waterlily flower color, some researchers have focused on the mechanism of blue flower formation [22, 26]. In addition to blue flowers, *N. atrans* and its hybrids can undergo petal color changes throughout the flowering period, resulting in higher ornamental value than others [27]. The *N*. ‘Feitian 2,’ intersubgeneric hybrid of *N. atrans*, perfectly inherits the characteristic of color-changing petals of *N. atrans*. In addition, its single flower possesses a longer flowering period, its stamen color is deep purple-red instead of yellow, and its petals are more discolored. Petals of *N*. ‘Feitian 2’ gradually change from light blue-purple to deep rose-red during flowering. However, the mechanism underlying this color change during flowering remains unclear.

To uncover this mechanism, *N*. ‘Feitian 2’, which changes petal color during flowering, was selected as the experimental subject. In the present study, we identified flavonoids, especially anthocyanins, in flower petals six days after opening via high-performance liquid chromatography (HPLC) and examined the relationship between flower coloration and pigment content. Moreover, transcriptomic analysis of petals from two different flowering stages was conducted to reveal the gene regulation responsible for the *N*. ‘Feitian 2’ color transition process. This research elucidates the mechanism of flower color change in waterlilies and guides the molecular breeding of ornamental plants.

## Materials and methods

### Plant materials

**Fig. 1:**
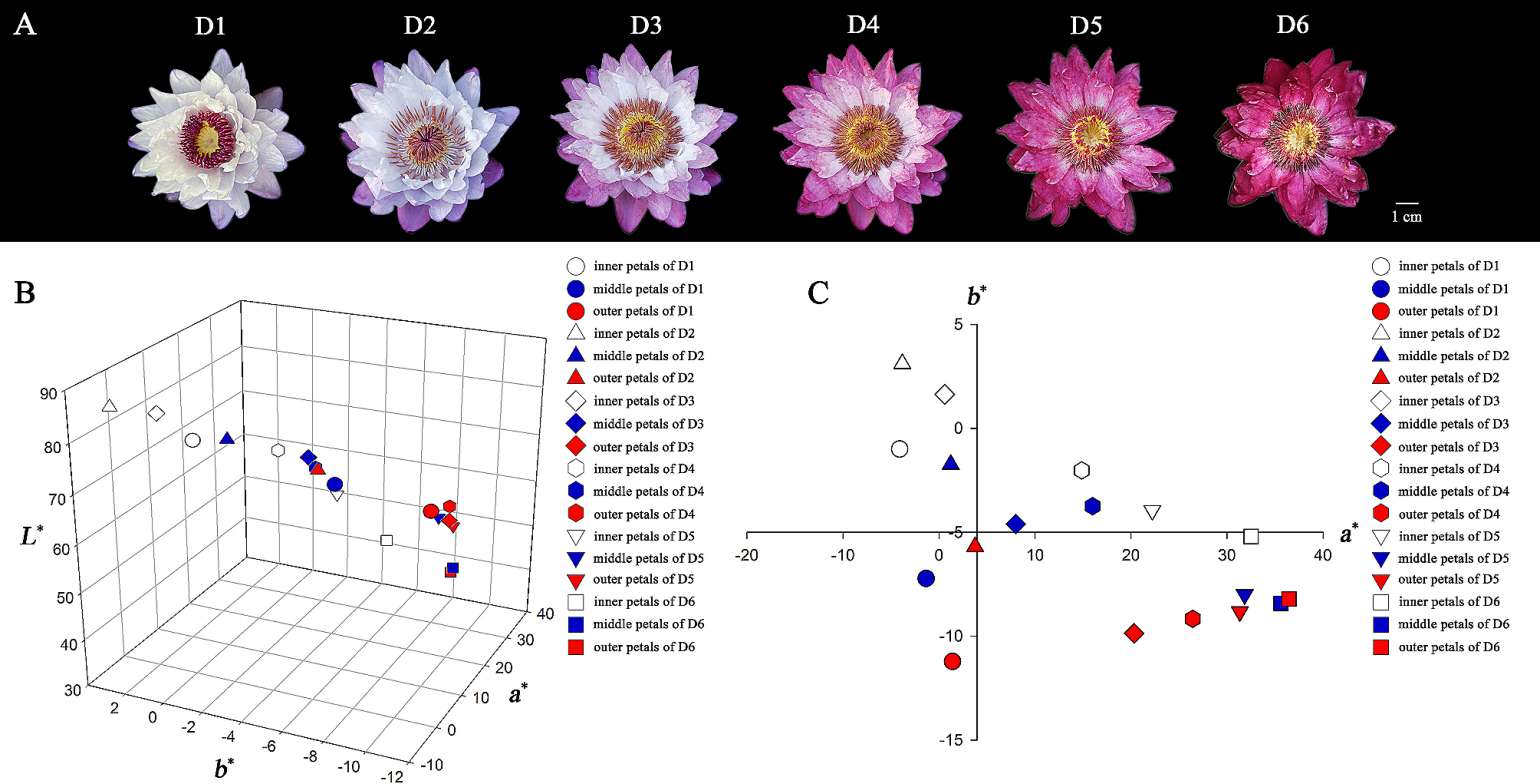
Various flowering stages and flower color distribution in coordinate systems of *N.* ‘Feitian 2’. **A,** Various flowering stages; **B,** flower color distribution across coordinate systems of trivariate (L^*^, a^*^, and b^*^); **C,**a^*^ and b^*^ value distribution during different flowering stages. (D1-D6 represents the first to the sixth blooming day, respectively)

The intersubgeneric day blooming waterlily cultivar *N.* ‘Feitian 2’ were used as materials in this study. This cultivar was a hybrid of subgenus *Anecphya* as female parent and subgenus *Brachyceras* as male parent. Single flower of *N.* ‘Feitian 2’ could bloom for 6 days (D1 to D6), and the color was different everyday (Fig. 1A). The petals (inner petals, middle petals, and outer petals) of *N.* ‘Feitian 2’ from D1 to D6 were collected at China National Botanical Garden/Institute of Botany, the Chinese Academy of Sciences, Beijing, China.

### Petal color measurement

The color parameters of fresh petals (inner petals, middle petals, and outer petals) were measured with a spectrophotometer NF555 (Nippon Denshoku Industries Co. Ltd., Japan) at CIE C/28 illumination/viewer conditions. CIELAB was used to measure different aspects of flower color by L^*^, a^*^, and b^*^ parameters. An average of five measurements was used. Then petals were snap-frozen in liquid nitrogen and stored at -80°C. Sampling was conducted with three biological replicates, to reduce analysis bias.

### Flavonoids extraction, qualitative and quantitative analysis

The flavonoids extraction of petals (D1 to D6, mixture of inner petals, middle petals, and outer petals) was carried out as described by a previous study, with minor modification [26]. First, samples were freeze-dried before the experiment. Then approximately 0.05 g of freeze-dried petals were pulverized in liquid nitrogen, extracted with 1 mL of extracting solution (99.8:0.2, v/v, methanol: formic acid) in a test tube, sonicated with KQ-500DE ultrasonic cleaner (Ultrasonic instruments, Jiangsu Kunshan, China) at 20°C for 20 min, and then centrifuged in SIGMA 3K30 (SIGMA centrifuger, Germany) with 10,000 g for 10 min. The supernatants were collected into fresh tubes. We repeated the above operation for three times. All extracts were combined and filtered through 0.22 μm reinforced nylon membrane filters (Shanghai ANPEL, Shanghai, China) before the HPLC-DAD and HPLC-MS analyses. Three replicates were made for each sample. All concentrations used in this study were calculated from dry weight (DW).

An Agilent 1260 Infinity II LC system (Agilent Technologies, Santa Clara, CA, USA) was used for the analysis. The HPLC analysis was performed under the following conditions: column, Kromasil 100-5 C_18_ column (250 mm × 4.6 mm; AKZO NOBEL, Sweden); solvent system, 2% formic acid aqueous solution (phase A) and 15% methanol acetonitrile (phase B); gradient program, 90:10 phase A/phase B at 0 min, 80:20 Phase A/phase B at 15.0 min, 77:23 phase A/phase B at 25.0 min, 60:40 phase A/phase B at 45.0 min, 10:90 phase A/phase B at 47.0 min, 10:90 phase A/phase B at 50.0 min; flow rate, 0.80 mL/min; temperature, 28 °C; injection volume: 10 µL. Chromatograms of anthocyanins and other flavonoids were acquired at 525 nm and 350 nm, respectively.

An Agilent 1260 Infinity II LC system coupled to an Agilent 6520 accurate-mass Q-TOF-MS/MS (Agilent Technologies, Santa Clara, CA, USA) was used for qualitative analysis. The liquid chromatographic conditions, mobile phase composition, and elution procedure were the same as those mentioned above. The following analysis conditions of mass spectrometry were adopted: the positive-ion (PI) mode for anthocyanins and negative-ion (NI) mode for other flavonoids; capillary voltage of 3.50 kV; nebulizer pressure of 0.103 MPa; desolvation gas (N_2_) flow of 12 L/min; drying gas temperature of 350°C; scanning range of 50-2000 (*m/z*) units. Data capture and analysis were managed using Masshunter Qualitative Analysis Software B.04.00.

The contents of anthocyanins and other flavonoids were calculated using semi-quantitative standard Cyanidin 3-*O*-glucoside (Cy3G) and rutin. Mean values and SDs were calculated from three biological replicates.

### RNA isolation, library construction and sequencing

Total RNA of D1 and D4 petals (mixture of inner petals, middle petals, and outer petals) was extracted using the E.Z.N.A. Plant RNA Kit (Omega Bio-tek, USA) according the instructions provided by the manufacturer. RNA integrity was assessed using the RNA Nano 6000 Assay Kit of the Agilent Bioanalyzer 2100 system (Agilent Technologies, CA, USA). RNA purity and concentration was measured using NanoDrop 2000 (Thermo Fisher Scientific, Wilmington, DE). A total amount of 1 µg RNA per sample was used as input material for the RNA sample preparations. Sequencing libraries were generated using Hieff NGS Ultima Dual-mode mRNA Library Prep Kit for Illumina (Yeasen Biotechnology (Shanghai) Co., Ltd.) following manufacturer’s recommendations and index codes were added to attribute sequences to each sample. Briefly, mRNA was isolated by Oligo (dT)-attached magnetic beads. Then mRNA was randomly fragmented into short fragments using fragmentation buffer and reverse transcribed into cDNA with random primers. Second-strand cDNA was synthesized with addition of PCR buffer, dNTPs, RNase H and DNA polymerase I. The cDNA fragments were purified with AMPure XP beads, end repaired, ‘A’ base added, and ligated to Illumina sequencing adapters. The ligation products were size selected by AMPure XP beads. In order to ensure the quality of library, Qubit 2.0 and Agilent 2100 were used to examine the concentration of cDNA and insert size. Q-PCR was processed to obtain a more accurate library concentration. At last, six libraries were sequenced on an Illumina NovaSeq platform to generate 150 bp paired-end reads, according to the manufacturer’s instructions (NCBI BioProject accession number: PRJNA1056490). The cDNA library construction and RNA-seq were performed by Biomarker Technologies Co., Ltd. (Beijing, China).

### Transcriptome data analysis

Clean reads were obtained by removing reads containing adapter, reads containing ploy-N and low quality reads from raw data. These clean reads were then mapped to the *N. colorata* reference genome using the default parameters of Hisat2 software [22]. Gene function was annotated based on the following databases: Nr (http://www.ncbi.nlm.nih.gov), Pfam (https://pfam.xfam.org/), KOG/COG (http://www.ncbi.nlm.nih.gov/COG), Swiss-Prot (https://www.expasy.org/), KEGG database (http://www.genome.jp/kegg), and GO (http://www.geneontology.org/). Fragments per kilobase of transcript per million mapped reads (FPKM) were used for transcription or quantification of gene expression levels. An absolute Log_2_FC ≥ 1 and false discovery rate (FDR) < 0.05 were used as thresholds for the identification of differentially expressed genes (DEGs) using DESeq2 software [28]. Gene Ontology (GO) enrichment analysis of the DEGs was implemented by the GOseq packages based Wallenius non-central hypergeometric distribution [29]. KOBAS database and clusterProfiler software were used to test the statistical enrichment of DEGs in KEGG pathways [30].

### RT-qPCR expression analysis of genes involved in flavonoids biosynthesis

For the purpose of gene validation and expression analysis, 16 candidate genes related to flavonoid biosynthesis were subjected to real-time quantitative PCR (RT-qPCR) on ABI StepOnePlus™ Real-Time PCR System (Applied Biosystems, USA). cDNA synthesis and RT-qPCR were performed using EX RT Kit (gDNAremover) and 2×HQ SYBR qPCR Mix (Zomanbio, Beijing, China). *Actin 11* was selected as an internal reference gene to normalize the expression data [31]. The gene-specific primers were shown in Supplementary Table S1. The 2^−ΔΔCT^ method was used to quantify gene expression [32].

### Correlation analysis of metabolite profiling and transcriptome

Mean and standard deviation for each sample were obtained from triplicate for further analysis. The heatmaps were performed by using TBtools [33]. Correlation analysis was performed by calculating the Pearson correlation coefficient (PCC with SPSS 21.0 (SPSS Inc., Chicago, IL)), and the screening criterion was PCC ≥ 0.80 or ≤ -0.80. Cytoscape (The Cytoscape Consortium, USA, version 3.9.1) was used to visualize the interaction networks.

## Results

### Petal color phenotype analysis of *Nymphaea* ‘Feitian 2’ at different flowering stages

A single flower of *Nymphaea* ‘Feitian 2’ bloomed for six days above the water. The color of petals differs across flowering stages; even different petals possess different levels of discoloration (Fig. 1A). In general, the outer petals change color first, while the inner petals change color beginning on day three after blooming (D3). Therefore, we divided the petals into inner petals, middle petals, and outer petals for color parameter determination. In the CIEL^*^a^*^b^*^ color system, the parameter L^*^ describes the lightness of the color, ranging from black (0) to white (100), the parameter a^*^ represents green and red color from negative value to positive value, and the parameter b^*^ represents blue and yellow color from negative value to positive value. The color coordinate values measured ranged across all petals as follows: L^*^ from 38.20 to 84.88, a^*^ from − 4.09 to 36.45, and b^*^ from − 11.22 to 3.11 (Supplementary Table S2). In general, a gradual discoloration process for *N.* ‘Feitian 2’ was observed, as its L^*^, a^*^, and b^*^ values were roughly distributed along a three-dimensional curve (Fig. 1B). The colors of different petals were generally consistent, owing to the a^*^ value and b^*^ value of both inner, middle, and outer petals being consistent (Fig. 1C).

### Identification and quantification of flavonoids

**Fig. 2:**
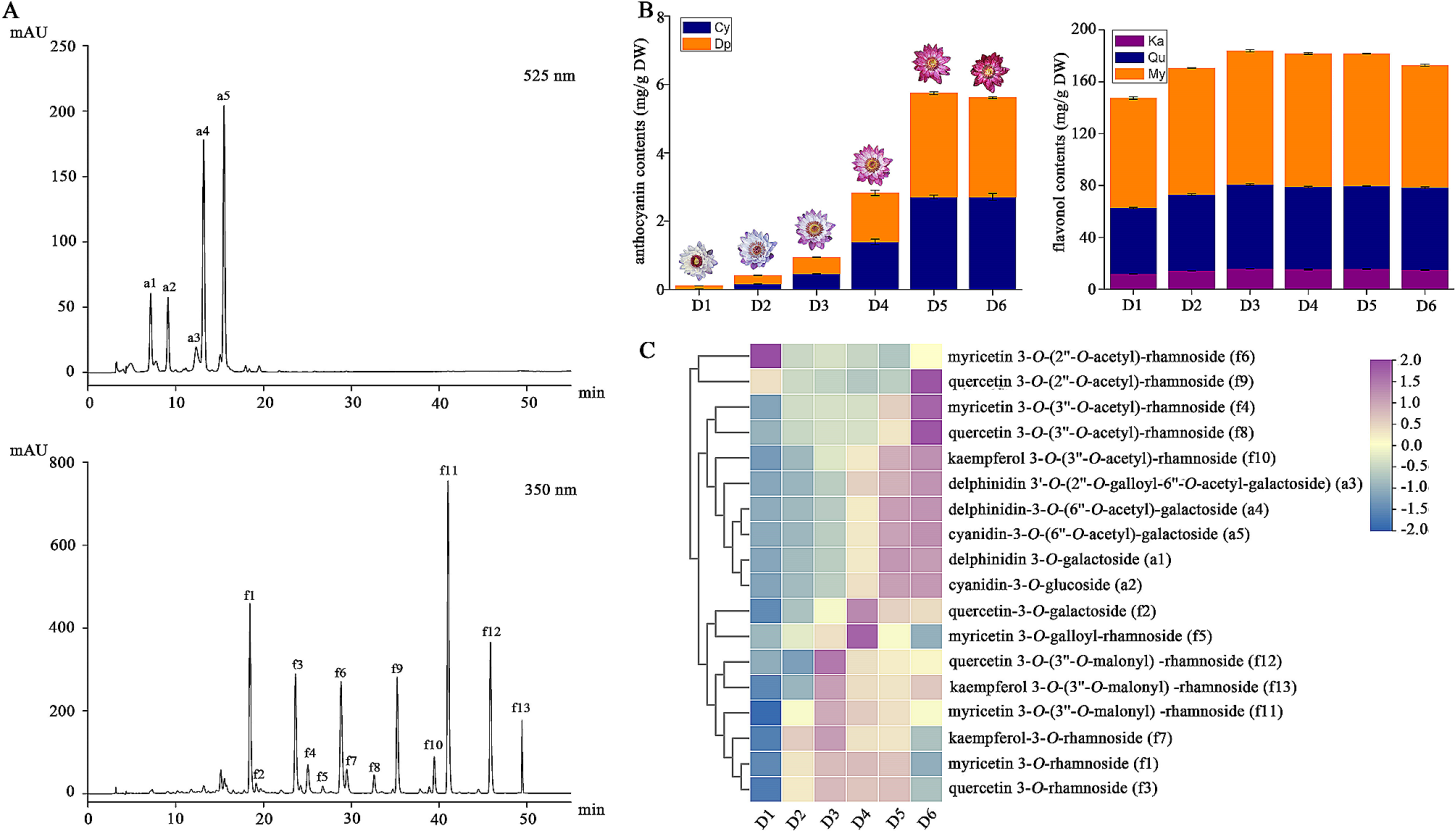
Analysis of flavonoid metabolites in the petals of *N.* ‘Feitian 2’. **A,** HPLC chromatograms of flavonoid detected at 525 nm and 350 nm. **B,** anthocyanin and flavonol content accumulation in petals, encompassing cyanidin derivatives (Cy), delphinidin derivatives (Dp), kaempferol derivatives (Ka) and quercetin derivatives (Qu), and myricetin derivatives (My). Three independent biological experiments were conducted. Values represent means ± SD. **C,** heatmap diagram of the 18 flavonoid metabolites across six flowering stages

We examined the flavonoid metabolites present in the petals of *N*. ‘Feitian 2’ across six different flowering stages (D1-D6) to determine the cause of petals becoming increasingly purple-red throughout flowering. A total of 18 flavonoid metabolites were identified, including five anthocyanins and 13 flavonols (Fig. 2A, Supplementary Table S3). The total anthocyanin contents (TA) accumulated constantly during anthesis over the first five days and decreased on the last day, with TA at D5 nearly 53 times higher than that at D1 (Fig. 2B, C, Supplementary Table S4). Only two aglycones of anthocyanidin, delphinidin and cyanidin, were identified. The levels of delphinidin derivatives were higher than cyanidin derivatives, which was the highest in D1, reaching 88.57% of TA, and then gradually decreased, reaching 50% of TA (Supplementary Table S4).

Flavonols, including kaempferol derivatives (Km), quercetin derivatives (Qu), and myricetin derivatives (My) were also identified. The total flavonol contents (TF) were approximately 147.46-183.88 mg g^− 1^ DW, and the highest TF level was detected at D3. The changing trend of TF from D1 to D6 showed a bell curve trend, which first increased and then fell. Myricetin derivatives represented the main components of flavonols, accounting for more than 50% of TF, followed by quercetin derivatives (34.41% of TF at D2 and 36.70% of TF at D6). The levels of kaempferol derivatives were lower in the petals (Supplementary Table S4). Unlike anthocyanins, the alterations in flavonol content across different flowering stages were complex. There were three types of change curves for flavonol contents, including an initial decrease followed by an increase, continuously increasing, and initially increasing and then decreasing (Fig. 2C).

Correlation analysis indicated that 10 flavonoids were significantly correlated with color parameters, including five anthocyanins and five flavonols (Supplementary Figure S1). Anthocyanins were significantly negatively correlated with the L^*^ value, positively correlated with the a^*^ value in both inner petals, middle petals and outer petals. And only negatively correlated with the b^*^ value in the inner petals. While there was no significant correlation with the middle or outer petals. The flavonols that were significantly correlated with color parameters were quercetin-3-*O*-galactoside (f2), myricetin 3-*O*-(3’’-*O*-acetyl)-rhamnoside (f4), quercetin 3-*O*-(3’’-*O*-acetyl)-rhamnoside (f8), kaempferol 3-*O*-(3’’-*O*-acetyl)-rhamnoside (f10), kaempferol 3-*O*-(3’’-*O*-malonyl) -rhamnoside (f13). Among them, f2 and f13 were only positively correlated with the a^*^ value of outer petals. In contrast, f4, f8, and f10 were negatively correlated with the L^*^ value and positively correlated with the a^*^ value of all petals.

According to the changes of flavonoids across different stages, all anthocyanins exhibited the largest changes in content. Delphinidin 3’-*O*-(2’’-*O*-galloyl-6’’-*O*-acetyl-galactoside) (a3) exhibited the lowest content among all anthocyanins, and its content increased nearly 5.74-fold, from 0.0629 mg g^− 1^ DW at D1 to 0.3612 mg g^− 1^ DW at D6 (Supplementary Table S4). In contrast, the five flavonols (f2, f4, f8, f10, and f13) were not changed as considerably as the anthocyanins. These results indicated that *N*. ‘Feitian 2’ flower color change is closely tied to the accumulation of five anthocyanins.

### RNA-Seq analysis

To delve deeper into the molecular mechanism of waterlily flower color changes during anthesis, six libraries (D1-1, D1-2, D1-3, D4-1, D4-2, and D4-3) were developed using *N.* ‘Feitian 2’ at two different flowering stages, replicated three times. After cleaning and quality control, approximately 64.36 Gb of clean data were obtained, and each library produced no less than 10.33 Gb of clean data. The Q30% of all libraries was over 92% (Supplementary Table S5). Between 62.92 and 64.73% of the sequenced reads could be aligned to the waterlily reference genome (Supplementary Table S5) [22]. Using an absolute Log_2_FC ≥ 1 and FDR < 0.01 as filter criteria for differentially expressed genes (DEGs), a total of 2,912 DEGs were found in D1 vs. D4, including 1,220 up-regulated DEGs and 1,692 down-regulated DEGs (Supplementary Figure S2). Therefore, a large number of genes may participate in petal color changing in *N*. ‘Feitian 2’.

To characterize the major functional categories of the DEGs, Gene Ontology (GO) enrichment analysis was performed. In total, 2,912 DEGs were distributed across three gene ontology categories: cellular component, biological process, and molecular function. In detail, the ‘metabolic process,’ ‘cellular process,’ and ‘single-organism process’ were the most enriched terms in biological processes. For the cellular component category, ‘membrane,’ ‘membrane part,’ and ‘cell’ were the most abundant proportions. Under the molecular function category, ‘catalytic activity,’ ‘binding,’ and ‘transporter activity’ were the most represented (Supplementary Figure S3).

**Fig. 3:**
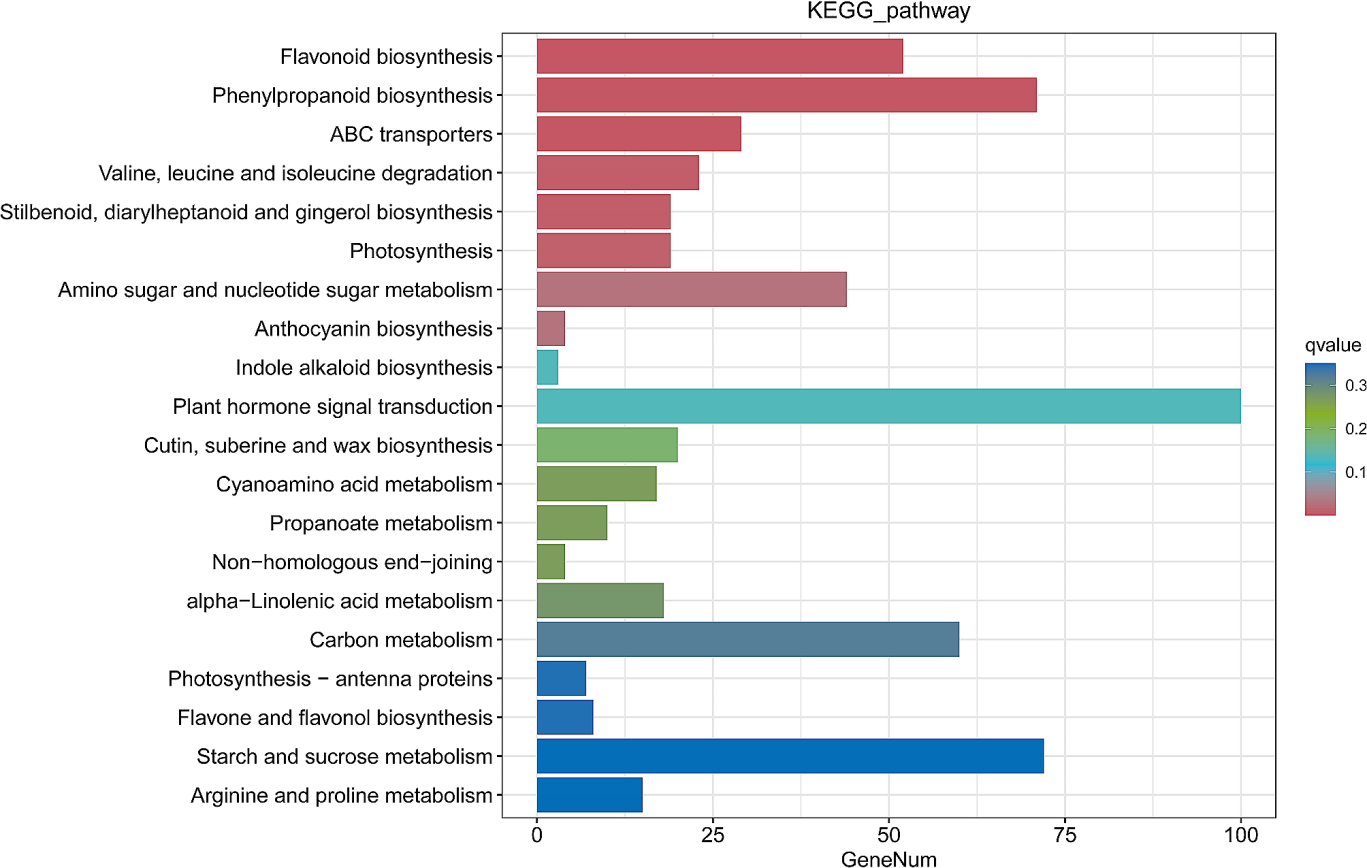
Bar chart depicting the top 20 genes analyzed using Kyoto Encyclopedia of Genes and Genomes (KEGG) terms enriched in differentially expressed genes (DEGs) in *N.* ‘Feitian 2’

Pathway analysis assists in understanding biological functions and gene interactions. Our findings demonstrated that pathways with the highest DEG representations were plant hormone signal transduction (ko00340), followed by starch and sucrose metabolism (ko00500) and phenylpropanoid biosynthesis (ko00940). The classification indicated that a large number of genes were enriched in ‘flavonoid biosynthesis’ (ko00941), ‘flavone and flavonol biosynthesis’ (ko00944), and ‘anthocyanin biosynthesis’ (ko00942), which were important for petal coloration (Fig. 3).

### Combined metabolomic and transcriptomic analysis

By integrating data from metabolomics and transcriptomics analyses, the flavonoid biosynthesis in *N.* ‘Feitian 2’ petals was revealed. We explored the genes of flavonoids, especially the anthocyanin biosynthesis pathway, and uncovered the key genes of flavonoid metabolism in the petals of *N.* ‘Feitian 2’ to analyze the mechanism of altered flower color. A total of 159 unigenes encoding nine enzymes involved in the above three pathways were the study focus (Table 1). We analyzed transcriptional profiles for the genes involved in flavonoid metabolism between D1 and D4 to identify the key genes for color changing. The results indicated that a total of 26 key unigenes not only had different expression levels but also FPKM values ≥ 10, including 19 up-regulated and seven down-regulated unigenes (Supplementary Table S6).

**Table 1:** Candidate genes associated with flavonoid biosynthesis in *N.* ‘Feitian 2’ petals

Gene	Enzyme	No. All^a^	No. DEGs^b^	No. Up^c^	No. Down^d^
*CHS*	Chalcone synthase	11	2	1	1
*CHI*	Chalcone isomerase	9	3	3	0
*F3H*	Flavanone 3-hydroxylase	8	3	1	2
*F3’H*	Flavonoid 3’-hydroxylase	12	1	1	0
*F3’5'H*	Flavonoid 3’5'-hydroxylase	3	2	2	0
*FLS*	Flavonol synthase	4	1	0	1
*DFR*	Dihydroflavonol 4-reductase	5	1	1	0
*ANS*	Anthocyanidin synthase	2	2	2	0
*UFGT*	Flavonoid 3-*O*-glucosyltransferase	105	11	8	3

No. All^a^, the total number of genes. No. DEGs^b^, the number of differentially expressed genes (DEGs) between two flowering satges. No. Up^c^, the number of up-regulated genes, and FPKM value ≥ 10. No. Down^d^, the number of down-regulated genes, and FPKM value ≥ 10

**Fig. 4:**
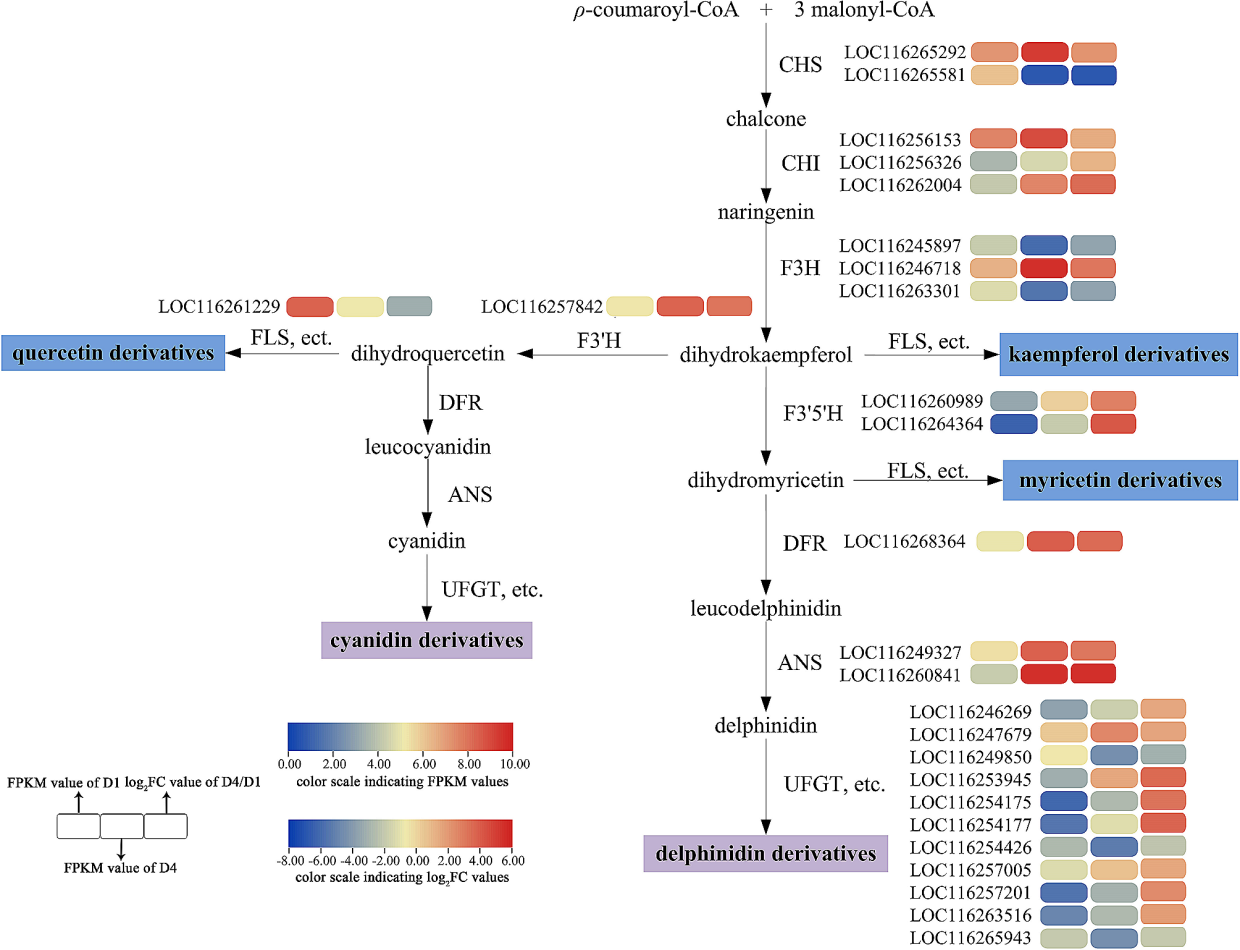
Evaluation of DEGs in the flavonoid biosynthesis pathway. CHS, chalcone synthase; CHI, chalcone isomerase; F3H, flavanone 3-hydroxylase; F3’H, flavonoid 3*'-*hydroxylase; F3’5'H, flavonoid 3’5'-hydroxylase; FLS, flavonol synthase; DFR, dihydroflavonol reductase; ANS, anthocyanidin synthase; UFGT, Flavonoid 3-*O*-glucosyltransferase

All identified DEGs contained both upstream genes (*CHS*, *CHI*, etc.) and downstream genes (*UFGT*, etc.) (Fig. 4). The majority of upstream genes, *CHS*, *CHI*, and *F3H*, were expressed higher at D4. However, *LOC116265581* (*CHS*-2), *LOC116245897* (*F3H*-1), and *LOC116263301* (*F3H*-3) had higher expression levels at D1. *F3’H*, *F3’5'H*, *DFR*, and *ANS* were all expressed more highly at D4, which was consistent with the accumulation of anthocyanins. *FLS* and *UFGT* were key enzyme genes for flavonoid biosynthesis and modification. Among them, the expression level of *LOC116261229* (*FLS*) was higher at D1, consistent with the trend of flavonol content. Additionally, *LOC116249850* (*UFGT*-3), *LOC116254426* (*UFGT*-7), and *LOC116265943* (*UFGT*-11), whose expression levels were higher at D1, might be the key genes for the modification of flavonol compounds. The expression of the remaining eight genes of *UFGT*s was higher at D4 than at D1, wherein *LOC116247679* (*UFGT-*2), *LOC116253945* (*UFGT-*4), and *LOC116257005* (*UFGT-*8) possessed higher FPKM values, and may represent the key unigenes related to anthocyanin modification. These suggested that the color alteration of waterlily petals was triggered by the co-expression of many unigenes.

**Fig. 5:**
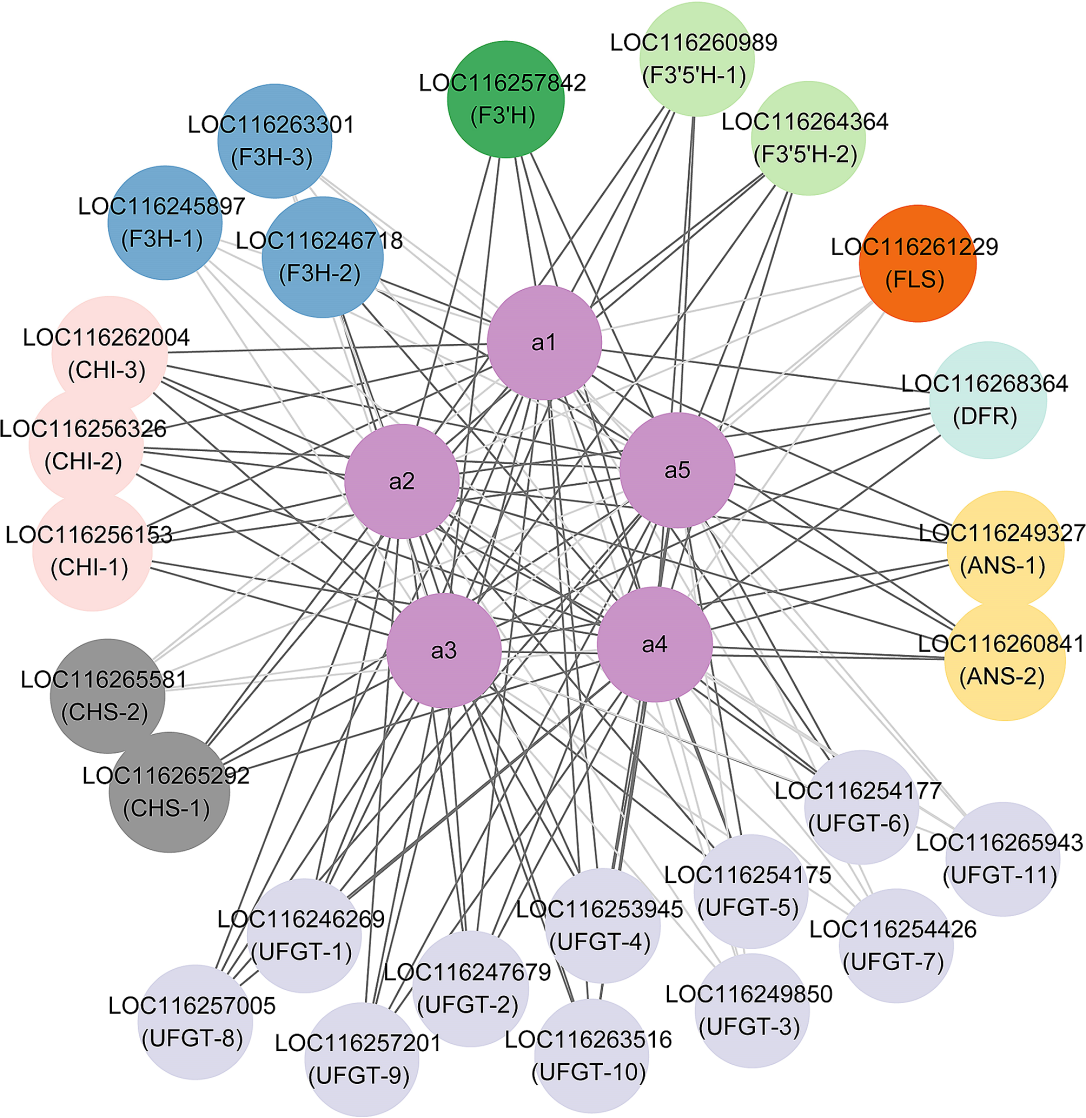
Co-expression network of DEGs in structure genes and flavonoid metabolism. The dark gray edges indicate a positive correlation, and the light gray indicates a negative correlation. a1, delphinidin 3-*O*-galactoside; a2, cyanidin-3-*O*-glucoside; a3, delphinidin 3’-*O*-(2’’-*O*-galloyl-6’’-*O*-acetyl-galactoside); a4, delphinidin-3-*O*-(6’’-*O*-acetyl)-galactoside; a5, cyanidin-3-*O*-(6’’-*O*-acetyl)-galactoside

Correlation analysis evaluated the correlation between the 26 DEGs mentioned above and five anthocyanins correlated with flower color change. Correlation analysis (Fig. 5) indicated that 19 DEGs were significantly positively correlated with five anthocyanins, including one *CHS*, three *CHI*, one *F3H*, one *F3’H*, two *F3’5'H*, one *DFR*, two *ANS*, and eight *UFGT*. In contrast, seven DEGs were significantly negatively correlated with five anthocyanins. After analyzing the expression levels of 19 positively correlated genes (Supplementary Table S6), we selected 12 candidate key structural genes, including *LOC116265292* (*CHS*-1), *LOC116256153* (*CHI*-1), *LOC116262004* (*CHI*-3), *LOC116246718* (*F3H*-2), *LOC116257842* (*F3’H*), *LOC116260989* (*F3’5'H*-1), *LOC116268364* (*DFR*), *LOC116249327* (*ANS*-1), *LOC116260841* (*ANS*-2), *LOC116247679* (*UFGT*-2), *LOC116253945* (*UFGT*-4), and *LOC116257005* (*UFGT*-8).

### RT-qPCR analysis

**Fig. 6:**
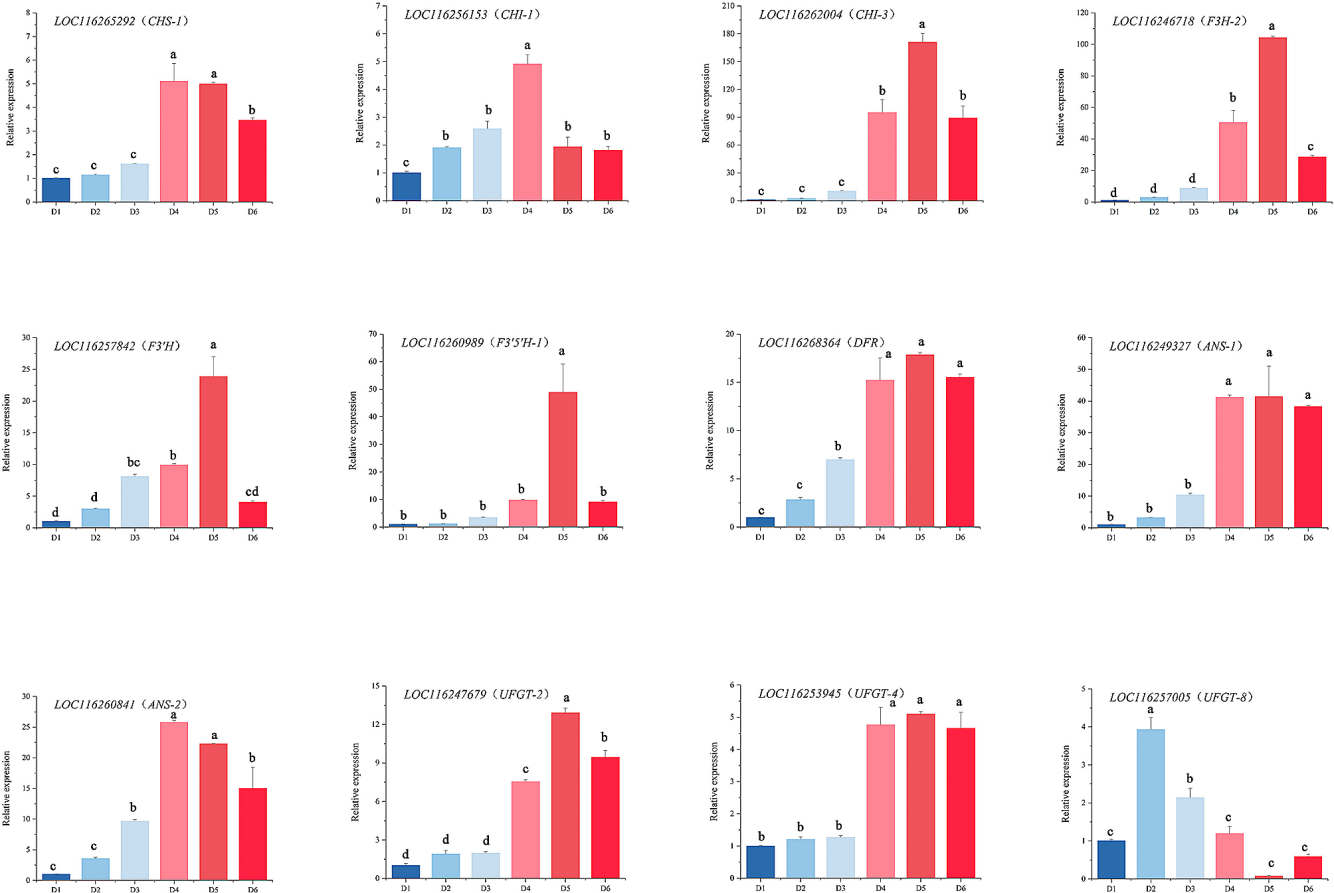
Relative expression levels of 12 candidate genes in the flavonoid biosynthetic pathway by RT-qPCR analysis. Data are presented as means ± SD

According to the correlation analysis using key flavonoids and expression level, 12 candidate enzyme genes were selected for RT-qPCR. The expression of these candidate genes increased continuously from D1 to D4 (Fig. 6), mirroring the trend measured in the transcriptome, indicating the accuracy of the sequencing data.

All 12 candidate genes had similar expression patterns, and their expression curves were “bell curves,” following an upward trend first, reaching the maximum level, and then exhibiting a downward trend. While the expression curves were similar, there were some differences. The expression levels of *LOC116265292* (*CHS*-1), *LOC116256153* (*CHI*-1), *LOC116249327* (*ANS*-1), and *LOC116260841* (*ANS*-2) were the highest at the D4 stage. The expression levels of *LOC116262004* (*CHI*-3), *LOC116246718* (*F3H*-2), *LOC116257842* (*F3’H*), *LOC116260989* (*F3’5'H*-1), *LOC116268364* (*DFR*), *LOC116247679* (*UFGT*-2), and *LOC116253945* (*UFGT*-4) were the highest at the D5 stage. The expression level of *LOC116257005* (*UFGT-*8) was the highest at the D2 stage. This indicates that *UFGT*-8 may be associated with flavonol synthesis. Among these genes, *CHI*-3, and *F3H*-2 had the most rapid change at different stages. Subsequently, *F3’H*, *F3’5'H*-1, *ANS*-1, and *ANS*-2 rapidly changed at different stages. *CHS*-1, *CHI*-1, *DFR*, *UFGT*-2, and *UFGT*-4 exhibited smaller changes during D1 to D6. The differential expression of structural genes could result in different flower colors of *N*. ‘Feitian 2’ across different flowering stages, among which the most important structural genes were *CHI*-3, *F3H*-2, *F3’H*, *F3’5'H*-1, *ANS*-1, and *ANS*-2.

### Specific transcription factor analysis

**Fig. 7:**
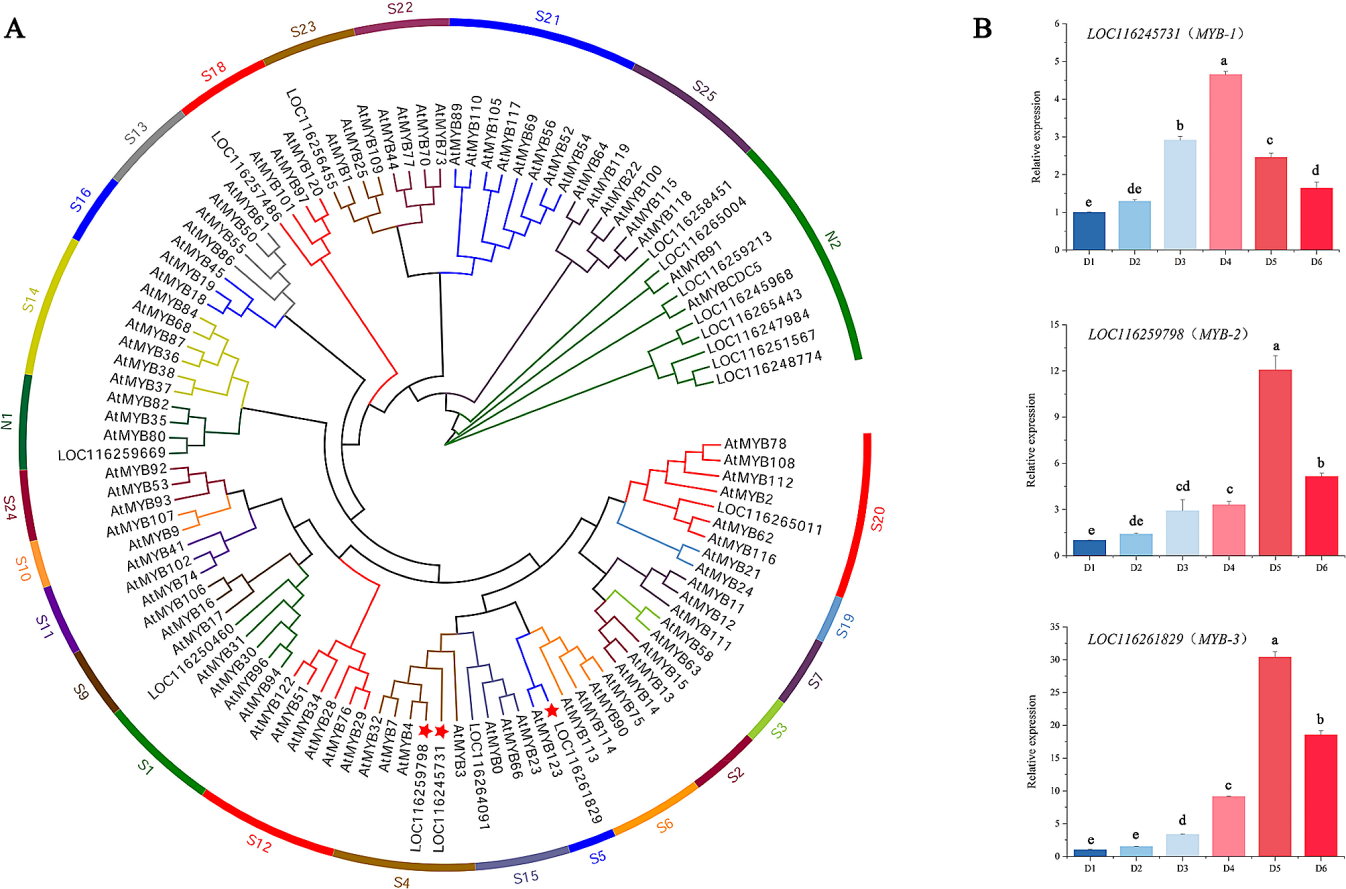
Phylogenetic tree and relative expression levels of *MYB*s derived from *N.* ‘Feitian 2’. **A,** phylogenetic tree of *MYBs* with *Arabidopsis*. **B,** relative expression levels of three *MYBs*

Transcription factors (TFs) execute critical roles in the growth and development of plants by modulating gene expression. Our data revealed a total of 1,208 unigenes predicted as TFs. A total of 104 transcription factors (FPKM value ≥ 10) with different expression levels were identified (Supplementary Table S7). Among these transcription factors, the most abundant were *MYB* (17), followed by *AP2*/*ERF* (14), *bHLH* (14), and *WRKY* (10). The TFs associated with regulating the expression of flavonoid structural genes were mainly MYB, bHLH, and WDR [34–37]. A total of 17 *MYB*, 14 *bHLH*, and 3 *WDR* unigenes were identified in our data. By constructing a phylogenetic tree alongside *Arabidopsis*, three *MYBs*, including *LOC116245731* (*MYB-1*), *LOC116259798* (*MYB-2*), and *LOC116261829* (*MYB-3*) were analyzed as crucial regulatory genes, as they were clustered together with *MYB*, related to the regulation of flavonoid biosynthesis in *Arabidopsis thaliana* (S4, S5, S6, and S7) (Fig. 7A) [38]. These three candidate regulatory genes were examined through RT-qPCR. These genes exhibited similar expression curves, known as a “bell curve” trend. The expression level of *LOC116245731* (*MYB*-*1*) was the highest at the D4 stage, however *LOC116259798* (*MYB*-*2*), and *LOC116261829* (*MYB*-*3*) were the highest at the D5 stage. *LOC116261829* (*MYB*-*3*) exhibited the most rapid change from D1 to D6 among three candidate regulatory genes. MYB may act as the mainly transcription factor in *N*. ‘Feitian 2’, as phylogenetic tree analysis identified that the candidate *bHLHs* did not converge with the S29 (IIIf subgroup), related to the regulation of flavonoid biosynthesis in *Arabidopsis thaliana* (Supplementary Figure S4) [39, 40].

## Discussion

Flavonoids, particularly anthocyanins, are vital in plant survival as attractants of pollinators and protectors in various stress situations [41]. To carry out their roles, they are typically produced transiently, undergoing regulated accumulation or degradation. Anthocyanin degradation causes color changes from deep to light, and the degradation mechanism is more comprehensively understood in *Brunfelsia calycina*, *Brunfelsia acuminata*, and *Nelumbo* ‘Qiusanse’ [9, 16, 42, 43]. Although much is known about anthocyanin biosynthesis, the mechanism of flower color change in each species differs, and as a basal angiosperm, the mechanism of flower color change in waterlilies remains unclear.

In many plants, the color of flower petals varies from light to deep, primarily due to the accumulation of anthocyanins. In *Rhododendron simsii*, for instance, the cyanidin biosynthesis pathway is activated to generate a red color [44]. Similarly, in the Japanese tree peony cultivar ‘Taiyoh,’ the pelargonidin biosynthesis pathway is activated in petals, resulting in a vivid red color [45]. In *Tulipa gesneiana* ‘Queen of night,’ the accumulation of anthocyanins (delphinidin 3-*O*-rutinoside, cyanidin 3-*O*-rutinoside, and pelargonidin 3-*O*-rutinoside) triggers a color change from green to black [46]. The shift in the waterlily cultivar ‘King of Siam’ petal in color from colorless to violet-blue throughout development is believed to have been caused by the cyanidin and delphinidin biosynthesis pathway [26]. Coloration operates as a gradual process, and many flowers have concluded coloration before opening, while some flowers undergo coloration after receiving certain signals, such as *Nymphaea* ‘Feitian 2,’ which begins coloration after opening. The anthocyanin accumulation in petals plays a crucial role in the coloration process. This study detected flavonoids in *N.* ‘Feitian 2’ petals over D1 to D6. A total of 18 flavonoids were characterized, including 13 flavonols and five anthocyanins (Table S3). Anthocyanins accumulated from D1 to D5, then reduced at D6, while flavonols accumulated from D1 to D3, then were reduced from D4 to D6 (Fig. 2). Only cyanidin derivatives and delphinidin derivatives were identified, which was consistent with the findings of other research [25, 47, 48]. While anthocyanidins are simple, glycosylation and acylation modification of anthocyanins were extensively present, producing rich and varied colors in waterlilies.

Anthocyanins are produced at the end of the phenylpropanoid metabolic pathway, and the precursors of anthocyanin biosynthesis are malonyl-CoA and coumaroyl-CoA. Most anthocyanins are synthesized via CHS and CHI condensation; F3H, F3’H, or F3’5'H oxidation; DFR and ANS/LDOX catalysis; GT, and AT modification [4, 6]. In our study, we conducted a transcriptome analysis of *N*. ‘Feitian 2’ at two stages (D1 and D4), and screened 26 enzyme genes that were significantly differentially expressed (Table 1; Fig. 4). Among these, 19 were up-regulated genes, and the remaining genes were down-regulated. DFR and ANS act as critical enzymes in the anthocyanin pathway that play an essential role in converting dihydroflavonol to anthocyanidins [49]. We identified five *DFR* genes, of which only one gene was up-regulated. Two ANS genes were detected, and all were up-regulated, aligned with the pattern of floral color development. Cyanidin and delphinidin undergo further glycosylation under the action of GTs to achieve stability [50]. Moreover, GTs make important contributions to flower color formation. The bicolor nature of the lotus ‘Dasajin’ is primarily caused by the defective accumulation of the gene *NnUFGT2* in the white portion of its petals, preventing the formation of glycosylated anthocyanins in the final metabolic step for flower color [51]. In *Lobelia erinus*, rhamnosylation is an essential process for lobelinin synthesis, and the expression of RT (ABTR2 and ABTR4) is required for the blue color of *Lobelia* flowers [52]. The formation of peony red flowers also relies on the action of GTs [45]. UFGT homologous unigenes were identified in *N*. ‘Feitian 2,’ with 11 differentially expressed, eight up-regulated, and three down-regulated (Table 1). Correlation analysis demonstrated that eight up-regulated unigenes had a positive correlation with flower color formation. Among them, *LOC116247679* (UFGT-2), *LOC116253945* (UFGT-4), and *LOC116257005* (UFGT-8) had higher FPKM values, which may be critical unigenes related to anthocyanin modification.

Anthocyanin biosynthesis is predominantly regulated by transcription factors at the transcriptional level. Currently, many kinds of transcription factors, including MYB, bHLH, WD40, DOF, MADS-box, and WRKY proteins, have been found to modulate anthocyanin biosynthesis [53–56]. Among them, MYB transcription factors exert a crucial influence on the regulation of flower color. MYB transcription factors can act independently. For instance, AtPAP1 and AtPAP2 both act as master regulators controlling anthocyanin biosynthesis in *Arabidopsis thaliana* [57, 58]. MYB TFs can also interact with other MYB transcription factors to carry out their functions. For example, PrMYBa1was found to activate PrF3H by interacting with PrMYBa2 to generate an ‘MM’ complex in red-purple blotches formation of *Paeonia rockii* ‘ShuShengPengMo’ [59]. In addition, MYB can bind to bHLH or create MBW complexes with bHLH and WDR. In *Actinidia chinensis*, the AcMYBF110-AcbHLH1-AcWDR1 complex directly targeted the promoters of anthocyanin synthetic genes to promote fruit color formation [60]. In this study, we examined the transcriptome data and found that 104 important transcription factors, including MYB, AP2/ERF, WRKY, bHLH, WD40, NAC, bZIP, and others, displayed significantly different expression levels between D1 and D4 (Supplementary Table S6). MYB transcription factors were chosen for phylogenetic analysis. *LOC116245731* (*MYB-1*), *LOC116259798* (*MYB-2*), and *LOC116261829* (*MYB-3*) were clustered together with MYB, which were associated with the regulation of flavonoid biosynthesis in *A. thaliana* (Fig. 7B) [38]. We speculated that these three transcription factors may be candidate regulators of anthocyanin biosynthesis in *N*. ‘Feitian’ flowers.

## Conclusions

In this study, flavonoids at different flowering stages and transcriptome data were utilized to reveal the discoloration of *N.* ‘Feitian 2’ petals. There were 18 flavonoids identified in the petals. The variation of the content of five detected anthocyanins was a chemical mechanism that contributed to the change in flower color. Moreover, a total of 26 differentially expressed genes (DEGs) of structural genes in the flavonoid biosynthesis pathway were uncovered. Among them, six structural genes were identified as candidate genes, as they were not only significantly positively correlated with anthocyanin accumulation, but also had rapid change during different flowering stages. Furthermore, 104 differentially expressed transcription factors (TFs) were identified, and three *MYBs* associated with flavonoid biosynthesis were screened via sequence phylogenetic analysis. These findings can help clarify the molecular mechanism and regulatory networks of flower discoloration in waterlilies and provide a biological basis for the breeding of novel cultivars.

### Electronic supplementary material

Below is the link to the electronic supplementary material.

Supplementary Material 1

Supplementary Material 2

Supplementary Material 3

Supplementary Material 4

Supplementary Material 5

Supplementary Material 6

Supplementary Material 7

Supplementary Material 8

Supplementary Material 9

Supplementary Material 10

Supplementary Material 11

## Data Availability

All relevant supporting data sets are included in the article and its supplemental files. The raw RNA-seq data have been submitted to the SRA database under accession number PRJNA1056490, and they can also be freely available at: https:// www. ncbi. nlm. nih. gov/sra/PRJNA1056490.

## Abbreviations

HPLC: High-performance liquid chromatography
FPKM: Fragments per kilobase of exon model per million mapped reads
FDR: False discovery rate
KEGG: Kyoto Encyclopedia of Genes and Genomes
GO: Gene Ontology
TA: Total anthocyanin contents
TF: Total flavonol contents
Cy: Cyanidin derivatives
Dp: Delphinidin derivatives
Km: Kaempferol derivatives
Qu: Quercetin derivatives
My: Myricetin derivatives
Cy3G: Cyanidin 3-*O*-glucoside
CHS: Chalcone synthase
CHI: Chalcone isomerase
F3H: Flavanone 3-hydroxylase
F3’H: Flavonoid 3’-hydroxylase
F3’5'H: Flavonoid 3’5'-hydroxylase
FLS: Flavonol synthase
DFR: Dihydroflavonol 4-reductase
ANS: Anthocyanidin synthase
UFGT: Flavonoid 3-*O*-glucosyltransferase
LDOX: Leucoanthocyanin dioxygenase
MYB: V-myb avian myeloblastosis viral oncogene homolog
bHLH: Basic helix-loop-helix
