# Supplementary material for: Integrative analysis of transcriptome and target metabolites uncovering flavonoid biosynthesis regulation of changing petal colors in Nymphaea ‘Feitian 2’
Source: BMC Plant Biol. 2024 May 7;24:370. doi: 10.1186/s12870-024-05078-5 (PMC11075258; doi:10.1186/s12870-024-05078-5)
Supplement: Supplementary file 1 — Supplementary Material 1 [file 12870_2024_5078_MOESM1_ESM.docx]

**Supplementary table S1. List of primers used for RT-qPCR analysis.**

| **Gene** | **Unigene id** | **Forward primer sequence (5'–3')** | **Reverse primer sequence (5'–3')** |
| --- | --- | --- | --- |
| *CHS-1* | *LOC116265292* | GCACAAGACCGAACTGAAGG | TGAGGTGGGTGATCTTGGAC |
| *CHI-1* | *LOC116256153* | CTGGAAGTCTGCTGGGATCT | TCTCAATCACTGCAACCCCT |
| *CHI-3* | *LOC116262004* | GGGATCGATTGGCAGCAATT | CTTCCTCTTTCCCCTCGGTT |
| *F3H-2* | *LOC116246718* | GGCCATTACCTTAGCAACGG | CATTATCGACGCCTCTCCCT |
| *F3'H* | *LOC116257842* | GGCTAAGAAGATGAAGGCGC | ATCGGTACCAGCTGTGAACA |
| *F3'5'H-1* | *LOC116260989* | CCCCAAGAACACTAGGCTCA | TTCCAGTCAAAGGCATGCAC |
| *DFR* | *LOC116268364* | AGGACCAAGAAGATGACGGG | TCATACTGGGAGGCATCGTC |
| *ANS-1* | *LOC116249327* | ACGAATGAGTACGGGAAGCA | GGGCACTTGGGGTAGTAGTT |
| *ANS-2* | *LOC116260841* | TCTTCCACCTCCTCTACCCA | ATGCCTCCCAGTTCCTTCTC |
| *UFGT-2* | *LOC116247679* | AGGGAGGAAGAACGACAAGG | TTCCTTGTGCTCCTCTACCG |
| *UFGT-4* | *LOC116253945* | CCTCGCAGTGTTCATAAGCC | CTCAACACTAGCCGGATTGC |
| *UFGT-8* | *LOC116257005* | GCCGCCAAATTCATGAGCTA | CCATTCCATAGCCCCTCCAT |
| *MYB1* | *LOC116245731* | CGAAGGAAGAAGATGAGCGC | TCGTCTGTGAAGTTGCCTCT |
| *MYB2* | *LOC116259798* | TGTTAAGCAGGGGTGTGGAT | GATGAGCTTGCTGAGAAGGC |
| *MYB3* | *LOC116261829* | CTTCATCATCGCCCACCAAG | CTTCTCTGGCTTTGGAACCG |
| *MYB4* | *LOC116264091* | TCGACTACATCCGAGAGCAC | GCAATCAACGACCACCTGTT |
| *Actin 11* |  | ATGTGGCACTGGACTATGAGC | AGAGTTGTAAGTGGTTTCGTGAAT |
