# Supplementary material for: Integrative analysis of transcriptome and target metabolites uncovering flavonoid biosynthesis regulation of changing petal colors in Nymphaea ‘Feitian 2’
Source: BMC Plant Biol. 2024 May 7;24:370. doi: 10.1186/s12870-024-05078-5 (PMC11075258; doi:10.1186/s12870-024-05078-5)
Supplement: Supplementary file 2 — Supplementary Material 2 [file 12870_2024_5078_MOESM2_ESM.docx]

**Supplementary table S2. Color parameters of petals.**

| Stage | inner petals | | | middle petals | | | outer petals | | |
| --- | --- | --- | --- | --- | --- | --- | --- | --- | --- |
|  | *L*^*^ | *a*^*^ | *b*^*^ | *L*^*^ | *a*^*^ | *b*^*^ | *L*^*^ | *a*^*^ | *b*^*^ |
| D1 | 81.260 | -4.094 | -1.000 | 75.836 | -1.334 | -7.230 | 72.278 | 1.410 | -11.222 |
| D2 | 84.888 | -3.822 | 3.114 | 79.544 | 1.222 | -1.738 | 75.068 | 3.762 | -5.692 |
| D3 | 82.740 | 0.602 | 1.636 | 74.878 | 8.002 | -4.602 | 59.738 | 20.332 | -9.868 |
| D4 | 71.442 | 14.872 | -2.018 | 68.546 | 16.000 | -3.742 | 59.108 | 26.418 | -9.164 |
| D5 | 59.998 | 22.218 | -3.942 | 53.030 | 31.834 | -7.996 | 52.010 | 31.334 | -8.820 |
| D6 | 45.398 | 32.504 | -5.204 | 39.912 | 35.618 | -8.422 | 38.200 | 36.446 | -8.212 |
