# Supplementary material for: Integrative analysis of transcriptome and target metabolites uncovering flavonoid biosynthesis regulation of changing petal colors in Nymphaea ‘Feitian 2’
Source: BMC Plant Biol. 2024 May 7;24:370. doi: 10.1186/s12870-024-05078-5 (PMC11075258; doi:10.1186/s12870-024-05078-5)
Supplement: Supplementary file 3 — Supplementary Material 3 [file 12870_2024_5078_MOESM3_ESM.docx]

**Supplementary table S3. Qualitative analysis of flavonoids.**

| **compound** | **Rt (min)** | **λmax (nm)** | **EST-PI MS/MS^2^ (*m/z*)** | **EST-NI MS/MS^2^ (*m/z*)** | **Identifacation** | **References** |
| --- | --- | --- | --- | --- | --- | --- |
| a1 | 7.143 | 277, 525 | 303[A+H] ^+^, 465[M+H]^+^ |  | delphinidin 3-*O*-galactoside | [1-4] |
| a2 | 9.120 | 280, 515 | 287[A+H]^+^, 449[M+H]^+^ |  | cyanidin-3-*O*-glucoside | standard |
| a3 | 12.327 | 282, 518 | 303[A+H]^+^, 659[M+H]^+^ |  | delphinidin 3'-*O*-(2''-*O*-galloyl-6''-*O*-acetyl-galactoside) | [3, 5, 6] |
| a4 | 13.176 | 276, 527 | 303[A+H]^+^, 507[M+H]^+^ |  | delphinidin-3-*O*-(6''-*O*-acetyl)-galactoside | [2, 4] |
| a5 | 15.496 | 282, 518 | 287[A+H]^+^, 491[M+H]^+^ |  | cyanidin-3-*O*-(6''-*O*-acetyl)-galactoside | [4] |
| f1 | 18.448 | 262, 350 | 319[A+H]^+^, 465[M+H]^+^ | 316[A-2H]^-^, 463[M-H]^-^ | myricetin 3-*O*-rhamnoside | [2-4, 7-10] |
| f2 | 19.154 | 255, 355 | 303[A+H]^+^, 465[M+H]^+^ | 300[A-2H]^-^, 463[M-H]^-^ | quercetin-3-*O*-galactoside | [4, 11] |
| f3 | 23.629 | 255, 346 | 303[A+H]^+^, 449[M+H]^+^ | 301[A-H]^-^, 447[M-H]^-^ | quercetin 3-*O*-rhamnoside | [3-8, 12] |
| f4 | 25.049 | 261, 348 | 319[A+H]^+^, 507[M+H]^+^ | 316[A-2H]^-^, 505[M-H]^-^ | myricetin 3-*O*-(3''-*O*-acetyl)-rhamnoside | [3, 6, 7, 10] |
| f5 | 26.745 | 265, 350 | 319[A+H]^+^, 465[M+H-152]^+^, 617[M+H]^+^ | 317[A-H]^-^, 463[M-H-152]^-^, 615[M-H]^-^ | myricetin 3-*O*-galloyl-rhamnoside | [5, 6] |
| f6 | 28.833 | 261, 348 | 319[A+H]^+^, 507[M+H]^+^ | 316[A-2H]^-^, 505[M-H]^-^ | myricetin 3-*O*-(2''-*O*-acetyl)-rhamnoside | [3, 5, 6] |
| f7 | 29.498 | 260, 345 | 287[A+H]^+^, 433[M+H]^+^ | 285[A-H]^-^, 431[M-H]^-^ | kaempferol-3-*O*-rhamnoside | [4, 5, 10, 12, 13] |
| f8 | 32.605 | 255, 348 | 303[A+H]^+^，491[M+H]^+^ | 300[A-2H]^-^，489[M-H]^-^ | quercetin 3-*O*-(3''-*O*-acetyl)-rhamnoside | [3, 5-7, 14] |
| f9 | 35.238 | 255, 350 | 303[A+H]^+^，491[M+H]^+^ | 300[A-2H]^-^，489[M-H]^-^ | quercetin 3-*O*-(2''-*O*-acetyl)-rhamnoside | [5, 6] |
| f10 | 39.458 | 263, 341 | 287[A+H]^+^，475[M+H]^+^ | 284[A-2H]^-^，473[M-H]^-^ | kaempferol 3-*O*-(3''-*O*-acetyl)-rhamnoside | [3, 5-7, 14] |
| f11 | 41.027 | 257, 348 | 319[A+H]^+^, 549[M+H]^+^ | 316[A-2H]^-^, 547[M-H]^-^ | myricetin 3-*O*-(3''-*O*-malonyl) -rhamnoside | [3] |
| f12 | 45.852 | 254, 347 | 303[A+H]^+^，533[M+H]^+^ | 300[A-2H]^-^，531[M-H]^-^ | quercetin 3-*O*-(3''-*O*-malonyl) -rhamnoside | [3] |
| f13 | 49.450 | 264, 341 | 287[A+H]^+^，517[M+H]^+^ | 284[A-2H]^-^，515[M-H]^-^ | kaempferol 3-*O*-(3''-*O*-malonyl) -rhamnoside | [3] |

**References**

1. Fossen T, Anderson ØM. Acylated anthocyanins from leaves of the water lily, *Nymphaéa* ×*marliacea*. Phytochemistry. 1997;46:353-357.

2. Fossen T, Larsenb A, Andersen ØM. Anthocyanins from flowers and leaves of *Nymphaéa* x *marliacea* cultivars. Phytochemistry. 1998b;48:823-827.

3. Wu Q, Wu J, Li SS, Zhang HJ, Feng CY, Yin DD, et al. Transcriptome sequencing and metabolite analysis for revealing the blue flower formation in waterlily. BMC Genomics. 2016;17:897.

4. Zhu ML, Wang LS, Zhang HJ, Xu YJ, Zheng XC, Wang LJ. Relationship between the composition of anthocyanins and flower color variation in hardy water lily（*Nymphaea* spp.）cultivars. Chinese Bulletin of Botany. 2012a;47:437-453.

5. Fossen T, Anderson ØM. Delphinidin 3’-galloylgalactosides from blue ﬂowers of *Nymphaéa* *caerulea*. Phytochemistry. 1999;50:1185-1188.

6. Zhu ML, Zheng XC, Shu QY, Li H, Zhong PX, Zhang HJ, et al. Relationship between the composition of flavonoids and flower colors variation in tropical water lily (*Nymphaea*) cultivars. PLoS One. 2012b;7:e34335.

7. Agnihotri VK, ElSohly HN, Khan SI, Smillie TJ, Khan IA, Walker LA. Antioxidant constituents of *Nymphaéa* *caerulea* flowers. Phytochemistry. 2008;69:2061-2066.

8. Elegami AA, Bates C, Gray AI, Mackay SP, Skellern GG, Waigh RD. Two very unusual macrocyclic flavonoids from the water lily *Nymphaea lotus*. Phytochemistry. 2003;63:727-731.

9. Fossen T, Frøystein NÅ, Andersen ØM. Myricetin 3-rhamnosyl (1→6) galactoside from *Nymphaéa* x *marliacea*. Phytochemistry. 1998a;49:1997-2000.

10. Zhang ZZ, ElSohly HN, Li XC, Khan SI, Jr. Broedel SE, Raulli RE, et al. Phenolic compounds from *Nymphaea odorata*. J Nat Prod. 2003;66:548-550.

11. Jambor J, Skrzypczak L. Flavonois from the flowers of *Nymphaea alba* L. ACTA SOC BOT POL. 1991;60:119-125.

12. Marquina S, Bonilla-Barbosa J, Alvarez L. Comparative phytochemical analysis of four Mexican *Nymphaea* species. Phytochemistry. 2005;66:921-927.

13. Liu RN, Wang W, Xie WD, Ding Y, Du LJ. *Nymphaea candida* flavonols: antioxidation and ischemic injury effect on neurons. World Science and Technology/Modernization of Traditional Chinese Medicine and Materia Medica. 2006;8:33-36.

14. Hsu CL, Fang SC, Yen GC. Anti-inflammatory effects of phenolic compounds isolated from the flowers of *Nymphaea mexicana* Zucc. FOOD FUNCT. 2013;4:1216.
