# Supplementary material for: Integrative analysis of transcriptome and target metabolites uncovering flavonoid biosynthesis regulation of changing petal colors in Nymphaea ‘Feitian 2’
Source: BMC Plant Biol. 2024 May 7;24:370. doi: 10.1186/s12870-024-05078-5 (PMC11075258; doi:10.1186/s12870-024-05078-5)
Supplement: Supplementary file 4 — Supplementary Material 4 [file 12870_2024_5078_MOESM4_ESM.docx]

**Supplementary table S4. Quantitative analysis of flavonoids.**

| Compound | Content (mg g^-1^ DW) | | | | | |
| --- | --- | --- | --- | --- | --- | --- |
|  | D1 | D2 | D3 | D4 | D5 | D6 |
| a1 | ND | 0.0179±0.0023 | 0.0468±0.0004 | 0.1876±0.0090 | 0.6739±0.0148 | 0.5239±0.0284 |
| a2 | ND | 0.0152±0.0004 | 0.0538±0.0008 | 0.2034±0.0121 | 0.5631±0.0152 | 0.4797±0.01980 |
| a3 | 0.0629±0.0009 | 0.0671±0.0052 | 0.0970±0.0041 | 0.2101±0.0272 | 0.3569±0.0471 | 0.3612±0.0335 |
| a4 | 0.0324±0.0005 | 0.1609±0.0017 | 0.3382±0.0055 | 1.0384±0.0660 | 2.0059±0.0478 | 2.0211±0.0847 |
| a5 | 0.0123±0.0004 | 0.1474±0.0048 | 0.4037±0.0057 | 1.1879±0.0671 | 2.1441±0.0393 | 2.2280±0.0784 |
| TA | 0.1076±0.0004 | 0.4085±0.0099 | 0.9396±0.0084 | 2.8274±0.1568 | 5.7439±0.0875 | 5.6138±0.1228 |
| f1 | 15.7369±0.2033 | 24.8291±0.0946 | 27.1423±0.1467 | 27.2432±0.0549 | 26.7007±0.2342 | 18.2617±0.1206 |
| f2 | 0.6676±0.0442 | 0.9086±0.0138 | 1.1483±0.0067 | 1.6457±0.2130 | 1.3932±0.0235 | 1.3838±0.0544 |
| f3 | 8.5580±0.0853 | 17.3044±0.2283 | 19.6700±0.3528 | 19.1701±0.1556 | 19.4021±0.1962 | 12.8347±0.2755 |
| f4 | 3.5963±0.0374 | 4.5110±0.0895 | 4.6059±0.0864 | 4.6468±0.1942 | 5.8110±0.1194 | 7.2775±0.1534 |
| f5 | 0.8489±0.0084 | 1.1431±0.1091 | 1.4127±0.0457 | 2.0553±0.0887 | 1.3108±0.0270 | 0.9288±0.0342 |
| f6 | 25.0429±0.2473 | 22.5544±0.0850 | 22.6920±0.1800 | 22.6127±0.2260 | 22.4039±0.3253 | 23.1929±0.3960 |
| f7 | 2.804±0.0297 | 4.8803±0.0607 | 5.3791±0.0417 | 4.7481±0.2438 | 4.7079±0.1515 | 3.7463±0.0843 |
| f8 | 1.8135±0.0201 | 2.3197±0.1531 | 2.4680±0.1034 | 2.5510±0.1126 | 3.2413±0.1129 | 4.9268±0.1349 |
| f9 | 19.1720±0.1935 | 17.7507±0.2485 | 17.6191±0.0408 | 17.4487±0.1322 | 17.5685±0.1562 | 21.8992±0.1740 |
| f10 | 4.7698±0.0413 | 4.9823±0.0785 | 5.3074±0.0493 | 5.6577±0.1142 | 5.9776±0.0735 | 6.1718±0.1229 |
| f11 | 39.6169±0.6130 | 44.6782±0.1469 | 47.3245±0.3972 | 46.4420±0.2188 | 45.8100±0.0416 | 44.8000±0.1474 |
| f12 | 20.6721±0.1834 | 20.4382±0.2817 | 24.1060±0.2458 | 22.6687±0.1487 | 22.4581±0.1941 | 22.3607±0.0339 |
| f13 | 4.1605±0.0390 | 4.3623±0.0593 | 5.0054±0.0782 | 4.8630±0.0656 | 4.8336±0.0504 | 4.9550±0.1132 |
| TF | 147.4596±1.4333 | 170.6624±0.9183 | 183.8807±1.4646 | 181.7530±1.5662 | 181.6187±0.5719 | 171.8293±1.5924 |
| ND means not detected this compound in flower petals. | | | |  |  |  |
