# Supplementary material for: Integrative analysis of transcriptome and target metabolites uncovering flavonoid biosynthesis regulation of changing petal colors in Nymphaea ‘Feitian 2’
Source: BMC Plant Biol. 2024 May 7;24:370. doi: 10.1186/s12870-024-05078-5 (PMC11075258; doi:10.1186/s12870-024-05078-5)
Supplement: Supplementary file 5 — Supplementary Material 5 [file 12870_2024_5078_MOESM5_ESM.docx]

**Supplementary table S5. RNA sequencing data and corresponding quality control.**

| **Sample** | **Clean Reads** | **Mapped Reads** | **Uniquely Mapped Reads** | **Multiple Mapped Reads** | **Q30 Percentage** |
| --- | --- | --- | --- | --- | --- |
| D1-1 | 75,714,078 | 48,722,540 (64.35%) | 47,315,525 (62.49%) | 1,407,015 (1.86%) | 94.75% |
| D1-2 | 69,182,310 | 43,933,866 (63.50%) | 42,717,488 (61.75%) | 1,216,378 (1.76%) | 92.52% |
| D1-3 | 77,029,818 | 49,858,518 (64.73%) | 48,432,252 (62.87%) | 1,426,266 (1.85%) | 94.77% |
| D4-1 | 70,015,924 | 44,705,353 (63.85%) | 43,498,414 (62.13%) | 1,206,939 (1.72%) | 95.20% |
| D4-2 | 69,024,630 | 43,430,987 (62.92%) | 42,254,210 (61.22%) | 1,176,777 (1.70%) | 93.42% |
| D4-3 | 69,222,866 | 43,641,920 (63.05%) | 42,422,805 (61.28%) | 1,219,115 (1.76%) | 92.86% |

Q30 percentage indicates the percentage of sequences with sequenceing error rate lower than 1‰
