# Supplementary material for: Integrative analysis of transcriptome and target metabolites uncovering flavonoid biosynthesis regulation of changing petal colors in Nymphaea ‘Feitian 2’
Source: BMC Plant Biol. 2024 May 7;24:370. doi: 10.1186/s12870-024-05078-5 (PMC11075258; doi:10.1186/s12870-024-05078-5)
Supplement: Supplementary file 6 — Supplementary Material 6 [file 12870_2024_5078_MOESM6_ESM.docx]

**Supplementary table S6. FPKM values of 26 flavonoid structural genes.**

| ID | Gene | D1 FPKM | D4 FPKM | FDR | log_2_FC |
| --- | --- | --- | --- | --- | --- |
| *LOC116265292* | *CHS-1* | 143.2454 | 643.5609 | 0.0000 | 2.1723 |
| *LOC116265581* | *CHS-2* | 68.0508 | 0.5595 | 0.0001 | -6.9044 |
| *LOC116256153* | *CHI-1* | 190.2796 | 477.6668 | 0.0000 | 1.3328 |
| *LOC116256326* | *CHI-2* | 10.8354 | 22.5155 | 0.0000 | 1.0583 |
| *LOC116262004* | *CHI-3* | 16.7731 | 182.5519 | 0.0000 | 3.4475 |
| *LOC116245897* | *F3H-1* | 17.7331 | 1.3987 | 0.0000 | -3.6476 |
| *LOC116246718* | *F3H-2* | 89.0095 | 784.0690 | 0.0000 | 3.1434 |
| *LOC116263301* | *F3H-3* | 25.0183 | 1.9047 | 0.0000 | -3.7023 |
| *LOC116257842* | *F3'H* | 36.3536 | 323.6343 | 0.0000 | 3.1583 |
| *LOC116260989* | *F3'5'H-1* | 7.6683 | 52.6224 | 0.0000 | 2.7824 |
| *LOC116264364* | *F3'5'H-2* | 0.9915 | 17.5259 | 0.0000 | 4.1350 |
| *LOC116268364* | *DFR* | 31.1586 | 351.6229 | 0.0000 | 3.5011 |
| *LOC116249327* | *ANS-1* | 41.0897 | 343.5952 | 0.0000 | 3.0688 |
| *LOC116260841* | *ANS-2* | 17.6380 | 794.8410 | 0.0000 | 5.4988 |
| *LOC116261229* | *FLS* | 330.9572 | 34.8992 | 0.0000 | -3.2403 |
| *LOC116246269* | *UFGT-1* | 6.6639 | 18.8070 | 0.0000 | 1.5010 |
| *LOC116247679* | *UFGT-2* | 60.4177 | 181.6507 | 0.0000 | 1.5929 |
| *LOC116249850* | *UFGT-3* | 35.4400 | 4.0554 | 0.0000 | -3.1221 |
| *LOC116253945* | *UFGT-4* | 8.9913 | 104.1375 | 0.0000 | 3.5380 |
| *LOC116254175* | *UFGT-5* | 1.2364 | 11.6195 | 0.0000 | 3.2338 |
| *LOC116254177* | *UFGT-6* | 2.0163 | 26.1240 | 0.0000 | 3.6952 |
| *LOC116254426* | *UFGT-7* | 11.4108 | 2.4048 | 0.0000 | -2.2361 |
| *LOC116257005* | *UFGT-8* | 24.7596 | 68.8978 | 0.0000 | 1.4809 |
| *LOC116257201* | *UFGT-9* | 1.8585 | 9.9383 | 0.0000 | 2.4219 |
| *LOC116263516* | *UFGT-10* | 3.2301 | 11.5645 | 0.0000 | 1.8450 |
| *LOC116265943* | *UFGT-11* | 17.0235 | 4.1028 | 0.0000 | -2.0456 |
