# Supplementary material for: Integrative analysis of transcriptome and target metabolites uncovering flavonoid biosynthesis regulation of changing petal colors in Nymphaea ‘Feitian 2’
Source: BMC Plant Biol. 2024 May 7;24:370. doi: 10.1186/s12870-024-05078-5 (PMC11075258; doi:10.1186/s12870-024-05078-5)
Supplement: Supplementary file 7 — Supplementary Material 7 [file 12870_2024_5078_MOESM7_ESM.docx]

**Supplementary table S7. Transcription factors of flavonoid biosynthesis.**

| id | TF_family | FPKM value | | log_2_FC |
| --- | --- | --- | --- | --- |
|  |  | D1 mean | D4 mean | D1 vs. D4 |
| LOC116246460 | AP2/ERF | 112.81 | 28.15 | -2.00 |
| LOC116248416 | AP2/ERF | 4.31 | 17.11 | 1.99 |
| LOC116251672 | AP2/ERF | 129.65 | 49.76 | -1.38 |
| LOC116252828 | AP2/ERF | 2.42 | 15.10 | 2.64 |
| LOC116256241 | AP2/ERF | 32.44 | 16.08 | -1.01 |
| LOC116259175 | AP2/ERF | 15.01 | 6.86 | -1.12 |
| LOC116260824 | AP2/ERF | 209.66 | 72.81 | -1.52 |
| LOC116261383 | AP2/ERF | 11.06 | 3.35 | -1.72 |
| LOC116261713 | AP2/ERF | 6.25 | 23.86 | 1.94 |
| LOC116265270 | AP2/ERF | 58.51 | 118.45 | 1.02 |
| LOC116265825 | AP2/ERF | 14.82 | 35.15 | 1.25 |
| LOC116265951 | AP2/ERF | 0.22 | 10.27 | 5.52 |
| LOC116266335 | AP2/ERF | 14.38 | 53.92 | 1.91 |
| LOC116267443 | AP2/ERF | 11.48 | 0.32 | -5.03 |
| LOC116266770 | ARF | 53.77 | 24.90 | -1.10 |
| LOC116252418 | B3 | 14.54 | 5.32 | -1.44 |
| LOC116261806 | B3 | 5.10 | 11.61 | 1.19 |
| LOC116247561 | bHLH | 9.61 | 24.71 | 1.37 |
| LOC116248240 | bHLH | 70.63 | 25.47 | -1.47 |
| LOC116248405 | bHLH | 55.88 | 13.82 | -2.01 |
| LOC116248711 | bHLH | 75.43 | 23.77 | -1.66 |
| LOC116251234 | bHLH | 113.98 | 371.38 | 1.71 |
| LOC116255522 | bHLH | 17.35 | 3.81 | -2.18 |
| LOC116256471 | bHLH | 29.96 | 6.02 | -2.31 |
| LOC116256545 | bHLH | 170.61 | 45.08 | -1.93 |
| LOC116256658 | bHLH | 3.92 | 12.92 | 1.72 |
| LOC116258120 | bHLH | 36.47 | 9.78 | -1.89 |
| LOC116259803 | bHLH | 1.96 | 10.56 | 2.43 |
| LOC116261572 | bHLH | 27.33 | 0.48 | -5.80 |
| LOC116262265 | bHLH | 117.07 | 28.77 | -2.02 |
| LOC116262863 | bHLH | 14.10 | 3.05 | -2.20 |
| LOC116253866 | bZIP | 18.18 | 5.09 | -1.83 |
| LOC116254126 | bZIP | 20.63 | 10.12 | -1.03 |
| LOC116256234 | bZIP | 24.23 | 11.57 | -1.06 |
| LOC116258933 | bZIP | 8.10 | 18.26 | 1.17 |
| LOC116259089 | bZIP | 8.14 | 51.64 | 2.67 |
| LOC116262068 | bZIP | 23.00 | 48.45 | 1.07 |
| LOC116250055 | C2H2 | 8.21 | 75.10 | 3.20 |
| LOC116252333 | C2H2 | 2.46 | 11.81 | 2.27 |
| LOC116266569 | C2H2 | 10.13 | 1.53 | -2.72 |
| LOC116254886 | CAMTA | 23.77 | 48.11 | 1.02 |
| LOC116262133 | CAMTA | 29.96 | 64.57 | 1.11 |
| LOC116259472 | CO-like | 19.26 | 38.73 | 1.01 |
| LOC116249081 | Dof | 22.62 | 53.97 | 1.26 |
| LOC116259722 | EIL | 101.28 | 50.28 | -1.01 |
| LOC116247606 | GATA | 34.93 | 16.64 | -1.06 |
| LOC116255584 | GATA | 16.01 | 3.45 | -2.21 |
| LOC116259927 | GATA | 13.09 | 35.75 | 1.45 |
| LOC116266325 | GATA | 40.58 | 8.95 | -2.18 |
| LOC116246060 | GRAS | 28.79 | 11.07 | -1.37 |
| LOC116249533 | GRAS | 102.70 | 2.22 | -4.76 |
| LOC116267088 | GRAS | 13.99 | 37.01 | 1.41 |
| LOC116248967 | HD-ZIP | 72.64 | 33.95 | -1.09 |
| LOC116253057 | HD-ZIP | 14.27 | 5.16 | -1.46 |
| LOC116255420 | HD-ZIP | 6.53 | 14.06 | 1.10 |
| LOC116259786 | HD-ZIP | 14.66 | 51.34 | 1.81 |
| LOC116263367 | HD-ZIP | 18.81 | 149.01 | 2.99 |
| LOC116265158 | HD-ZIP | 165.11 | 72.76 | -1.18 |
| LOC116252265 | LBD | 4.38 | 10.30 | 1.24 |
| LOC116265777 | MIKC_MADS | 2640.09 | 499.73 | -2.40 |
| LOC116266920 | MIKC_MADS | 14.52 | 45.10 | 1.63 |
| LOC116267701 | MIKC_MADS | 17.16 | 5.56 | -1.62 |
| LOC116245731 | MYB | 17.50 | 76.64 | 2.14 |
| LOC116245968 | MYB | 8.47 | 17.16 | 1.05 |
| LOC116247984 | MYB | 7.43 | 17.52 | 1.25 |
| LOC116248774 | MYB | 12.26 | 4.47 | -1.45 |
| LOC116250460 | MYB | 16.96 | 2.57 | -2.71 |
| LOC116251567 | MYB | 94.40 | 45.79 | -1.04 |
| LOC116256455 | MYB | 5.86 | 18.53 | 1.67 |
| LOC116257486 | MYB | 96.49 | 41.57 | -1.21 |
| LOC116258451 | MYB | 0.53 | 19.24 | 5.10 |
| LOC116259213 | MYB | 0.00 | 17.89 | 11.81 |
| LOC116259669 | MYB | 6.24 | 16.84 | 1.44 |
| LOC116259798 | MYB | 25.09 | 66.10 | 1.40 |
| LOC116261829 | MYB | 4.06 | 21.54 | 2.41 |
| LOC116264091 | MYB | 7.29 | 25.97 | 1.84 |
| LOC116265004 | MYB | 19.64 | 73.46 | 1.91 |
| LOC116265011 | MYB | 29.02 | 504.45 | 4.13 |
| LOC116265443 | MYB | 10.79 | 5.16 | -1.05 |
| LOC116245095 | NAC | 10.85 | 22.54 | 1.06 |
| LOC116249017 | NAC | 5.20 | 32.16 | 2.63 |
| LOC116254973 | NAC | 10.61 | 89.93 | 3.09 |
| LOC116258155 | NAC | 25.86 | 136.74 | 2.41 |
| LOC116264933 | NAC | 17.50 | 70.10 | 2.01 |
| LOC116267167 | NAC | 41.44 | 88.94 | 1.11 |
| LOC116267783 | NAC | 38.30 | 169.00 | 2.15 |
| LOC116262800 | SRS | 5.69 | 15.53 | 1.45 |
| LOC116248024 | TALE | 34.63 | 218.82 | 2.67 |
| LOC116249192 | TCP | 6.82 | 32.83 | 2.27 |
| LOC116257000 | TCP | 7.74 | 23.89 | 1.63 |
| LOC116248261 | WDR | 6.75 | 29.94 | 2.15 |
| LOC116258338 | WDR | 4.92 | 38.40 | 2.96 |
| LOC116266589 | WDR | 13.49 | 1.25 | -3.43 |
| LOC116246167 | WRKY | 32.52 | 77.54 | 1.26 |
| LOC116249634 | WRKY | 37.28 | 148.58 | 2.00 |
| LOC116253885 | WRKY | 2.55 | 10.55 | 2.04 |
| LOC116255078 | WRKY | 24.74 | 54.82 | 1.15 |
| LOC116258146 | WRKY | 12.81 | 73.96 | 2.53 |
| LOC116259969 | WRKY | 5.91 | 18.36 | 1.65 |
| LOC116261711 | WRKY | 14.49 | 33.83 | 1.23 |
| LOC116262672 | WRKY | 27.64 | 56.74 | 1.04 |
| LOC116265008 | WRKY | 17.82 | 75.42 | 2.09 |
| LOC116266640 | WRKY | 3.80 | 19.40 | 2.36 |
| LOC116262892 | ZF-HD | 7.33 | 1.48 | -2.28 |
