# Supplementary material for: Integrative analysis of transcriptome and target metabolites uncovering flavonoid biosynthesis regulation of changing petal colors in Nymphaea ‘Feitian 2’
Source: BMC Plant Biol. 2024 May 7;24:370. doi: 10.1186/s12870-024-05078-5 (PMC11075258; doi:10.1186/s12870-024-05078-5)
Supplement: Supplementary file 8 — Supplementary Material 8 [file 12870_2024_5078_MOESM8_ESM.pdf]

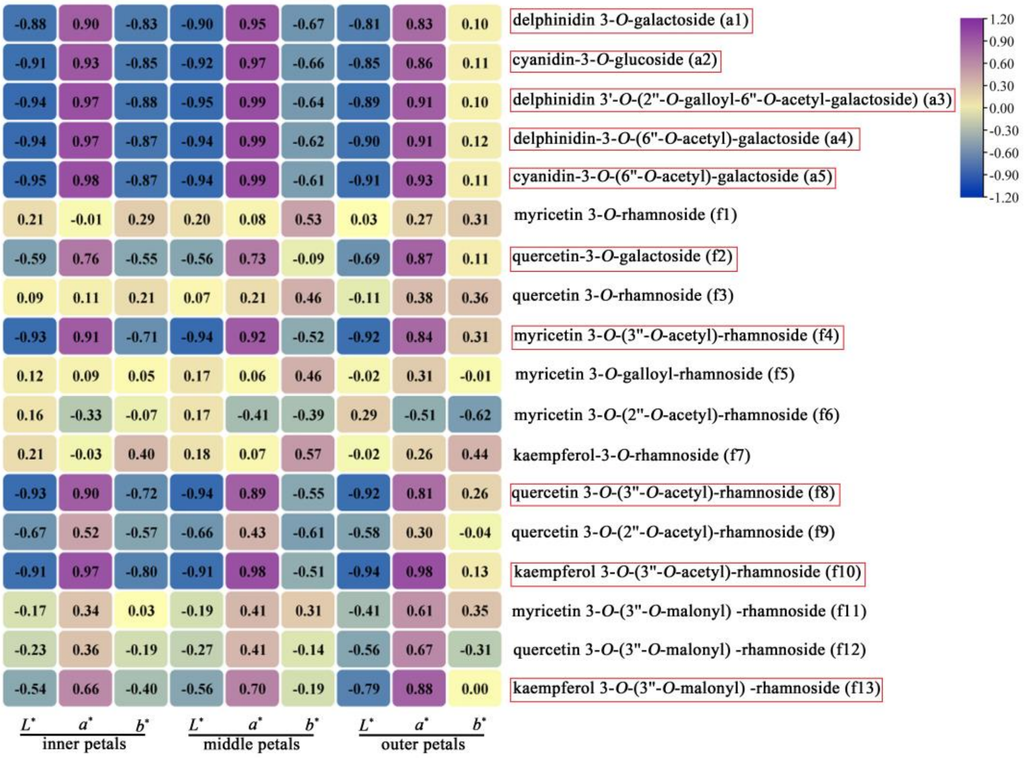

Supplementary Figure S1. Correlation analysis between color parameters and flavonoid components.
