# Supplementary figures and images for: Integrative analysis of transcriptome and target metabolites uncovering flavonoid biosynthesis regulation of changing petal colors in Nymphaea ‘Feitian 2’
Source: BMC Plant Biol. 2024 May 7;24:370. doi: 10.1186/s12870-024-05078-5 (PMC11075258; doi:10.1186/s12870-024-05078-5)

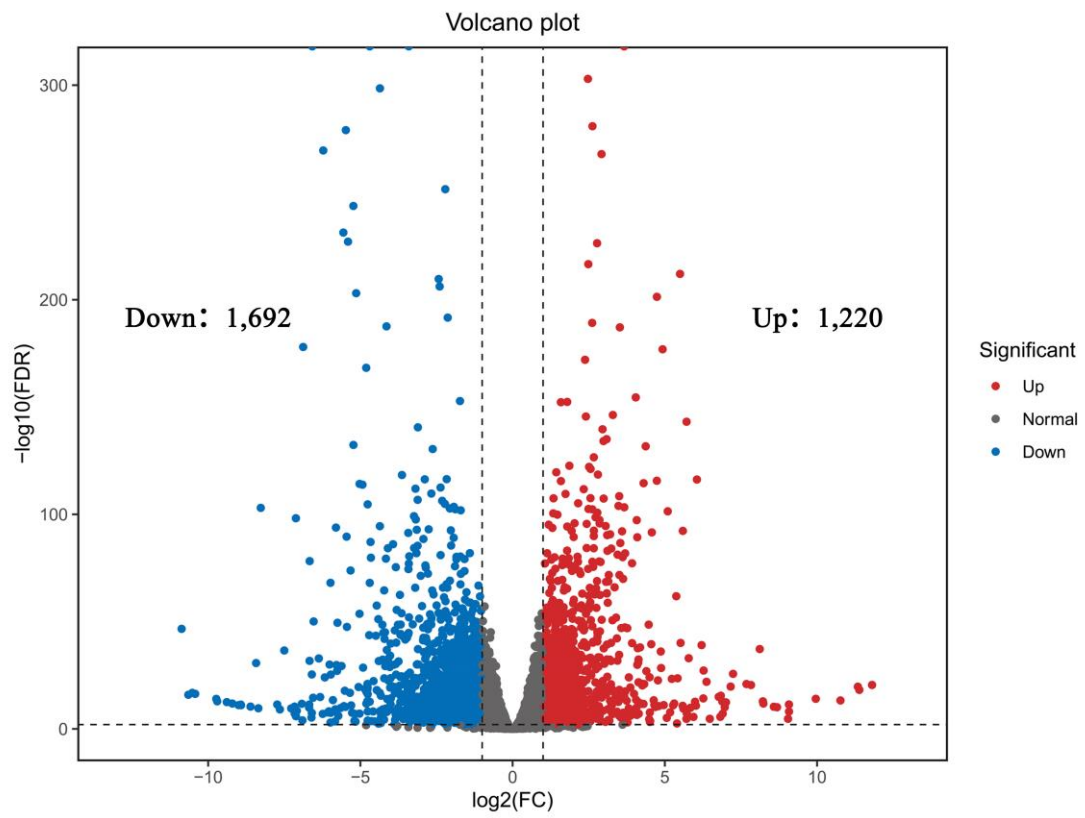

**Supplementary Figure S2. Volcano plots of differentially expressed genes (DEGs).**

Supplement: Supplementary file 9 — Supplementary Material 9 [file 12870_2024_5078_MOESM9_ESM.pdf]

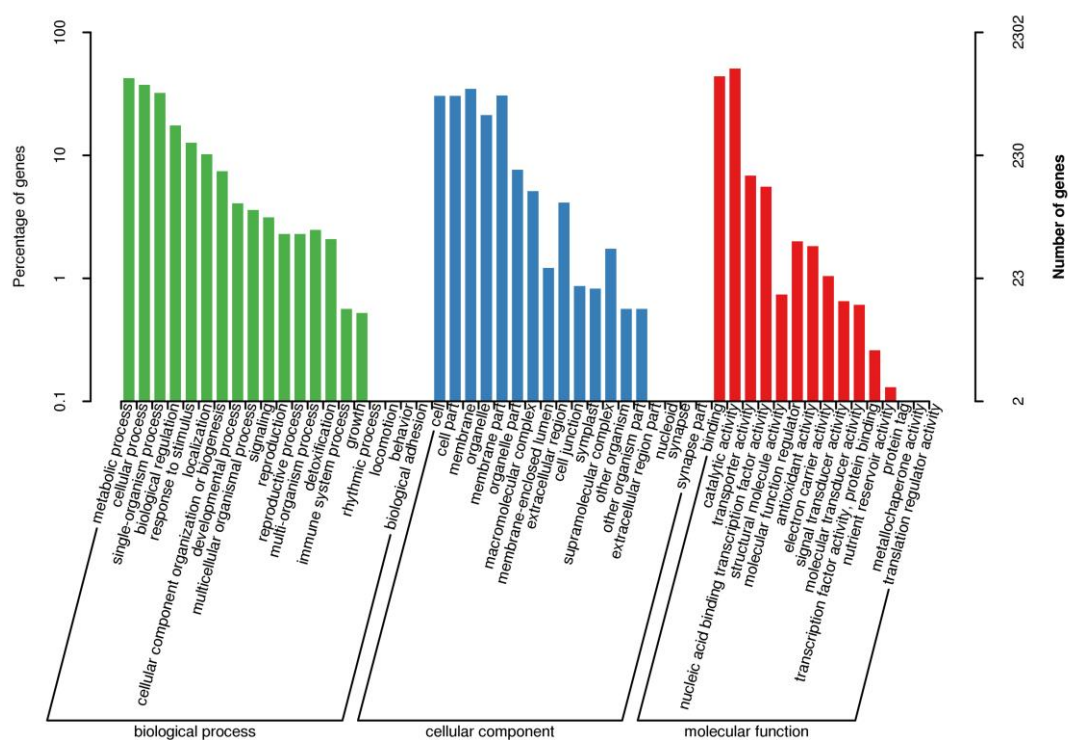

**Supplementary Figure S3. GO functional classification of DEGs.**

Supplement: Supplementary file 10 — Supplementary Material 10 [file 12870_2024_5078_MOESM10_ESM.pdf]

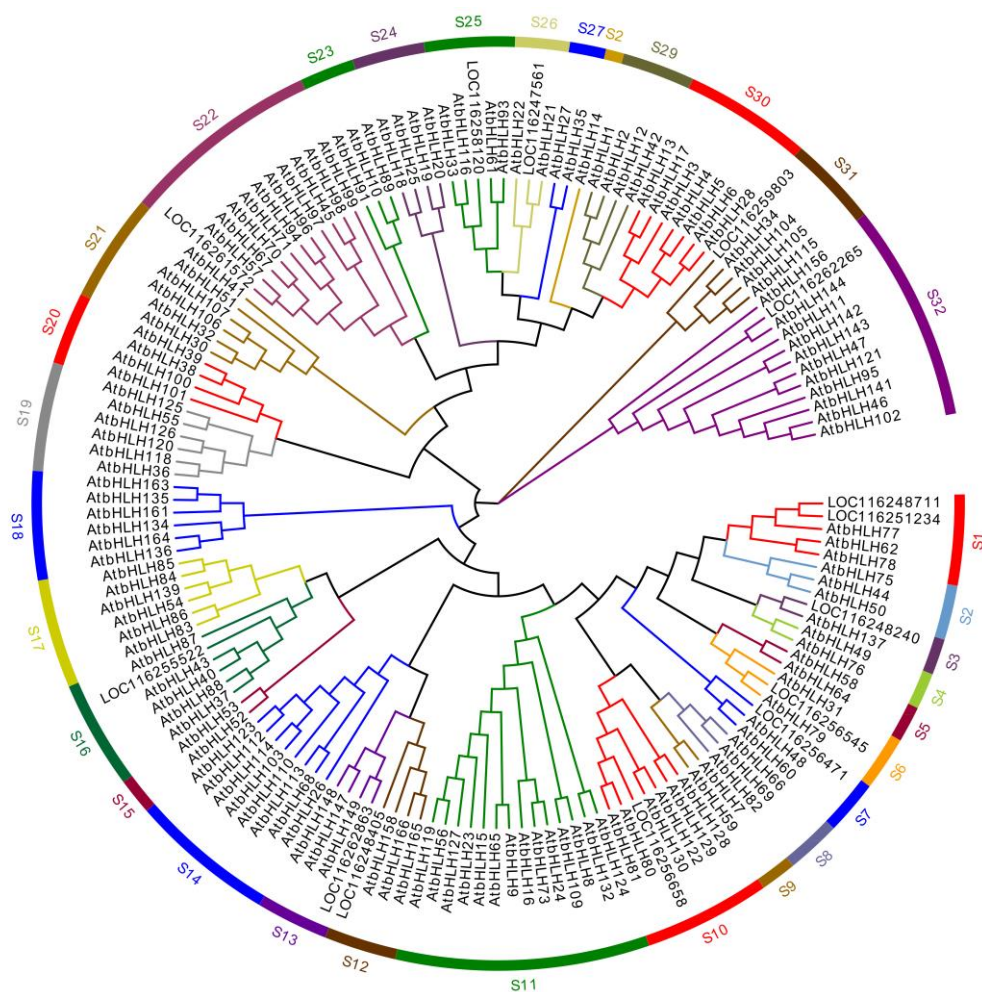

**Supplementary Figure S4. Phylogenetic tree of bHLHs from *N. 'Feitian 2'* and *Arabidopsis*.**

Supplement: Supplementary file 11 — Supplementary Material 11 [file 12870_2024_5078_MOESM11_ESM.pdf]
